# TMEM63C mutations cause mitochondrial morphology defects and underlie hereditary spastic paraplegia

**DOI:** 10.1093/brain/awac123

**Published:** 2022-06-18

**Authors:** Luis Carlos Tábara, Fatema Al-Salmi, Reza Maroofian, Amna Mohammed Al-Futaisi, Fathiya Al-Murshedi, Joanna Kennedy, Jacob O Day, Thomas Courtin, Aisha Al-Khayat, Hamid Galedari, Neda Mazaheri, Margherita Protasoni, Mark Johnson, Joseph S Leslie, Claire G Salter, Lettie E Rawlins, James Fasham, Almundher Al-Maawali, Nikol Voutsina, Perrine Charles, Laura Harrold, Boris Keren, Edmund R S Kunji, Barbara Vona, Gholamreza Jelodar, Alireza Sedaghat, Gholamreza Shariati, Henry Houlden, Andrew H Crosby, Julien Prudent, Emma L Baple

**Affiliations:** Medical Research Council Mitochondrial Biology Unit, University of Cambridge, Cambridge CB2 0XY, UK; Level 4, RILD Wellcome Wolfson Medical Research Centre, RD&E (Wonford) NHS Foundation Trust, University of Exeter Medical School, Exeter EX2 5DW, UK; UCL Queen Square Institute of Neurology, University College London, London WC1E 6BT, UK; Genetic and Developmental Medicine Clinic, Department of Genetics, College of Medicine and Health Sciences, Sultan Qaboos University Hospital, Muscat 123, Oman; Genetic and Developmental Medicine Clinic, Department of Genetics, College of Medicine and Health Sciences, Sultan Qaboos University Hospital, Muscat 123, Oman; Level 4, RILD Wellcome Wolfson Medical Research Centre, RD&E (Wonford) NHS Foundation Trust, University of Exeter Medical School, Exeter EX2 5DW, UK; Clinical Genetics, University Hospitals Bristol, Bristol BS2 8EG, UK; Level 4, RILD Wellcome Wolfson Medical Research Centre, RD&E (Wonford) NHS Foundation Trust, University of Exeter Medical School, Exeter EX2 5DW, UK; Faculty of Health, University of Plymouth, Plymouth PL4 8AA, UK; Département de génétique, Hôpital Pitié-Salpêtrière, Assistance Publique-Hôpitaux de Paris, 75019 Paris, Sorbonne Université, France; Department of Biology, College of Science, Sultan Qaboos University, Muscat, Oman; Department of Genetics, Faculty of Science, Shahid Chamran University of Ahvaz, Ahvaz, Iran; Department of Genetics, Faculty of Science, Shahid Chamran University of Ahvaz, Ahvaz, Iran; Medical Research Council Mitochondrial Biology Unit, University of Cambridge, Cambridge CB2 0XY, UK; Medical Research Council Mitochondrial Biology Unit, University of Cambridge, Cambridge CB2 0XY, UK; Level 4, RILD Wellcome Wolfson Medical Research Centre, RD&E (Wonford) NHS Foundation Trust, University of Exeter Medical School, Exeter EX2 5DW, UK; Level 4, RILD Wellcome Wolfson Medical Research Centre, RD&E (Wonford) NHS Foundation Trust, University of Exeter Medical School, Exeter EX2 5DW, UK; Level 4, RILD Wellcome Wolfson Medical Research Centre, RD&E (Wonford) NHS Foundation Trust, University of Exeter Medical School, Exeter EX2 5DW, UK; Peninsula Clinical Genetics Service, Royal Devon and Exeter Hospital (Heavitree), Exeter EX1 2ED, UK; Level 4, RILD Wellcome Wolfson Medical Research Centre, RD&E (Wonford) NHS Foundation Trust, University of Exeter Medical School, Exeter EX2 5DW, UK; Peninsula Clinical Genetics Service, Royal Devon and Exeter Hospital (Heavitree), Exeter EX1 2ED, UK; Genetic and Developmental Medicine Clinic, Department of Genetics, College of Medicine and Health Sciences, Sultan Qaboos University Hospital, Muscat 123, Oman; Level 4, RILD Wellcome Wolfson Medical Research Centre, RD&E (Wonford) NHS Foundation Trust, University of Exeter Medical School, Exeter EX2 5DW, UK; Département de génétique, Hôpital Pitié-Salpêtrière, Assistance Publique-Hôpitaux de Paris, 75019 Paris, Sorbonne Université, France; Level 4, RILD Wellcome Wolfson Medical Research Centre, RD&E (Wonford) NHS Foundation Trust, University of Exeter Medical School, Exeter EX2 5DW, UK; Département de génétique, Hôpital Pitié-Salpêtrière, Assistance Publique-Hôpitaux de Paris, 75019 Paris, Sorbonne Université, France; Medical Research Council Mitochondrial Biology Unit, University of Cambridge, Cambridge CB2 0XY, UK; Department of Otolaryngology-Head and Neck Surgery, Tübingen Hearing Research Centre, Eberhard Karls University Tübingen, Tübingen, Germany; Pediatric Neurology, Ahvaz Jundishapur University of Medical Sciences, Ahvaz, Iran; Health Research Institute, Diabetes Research Center, Ahvaz Jundishapur University of Medical Sciences, Ahvaz, Iran; Department of Medical Genetic, Faculty of Medicine, Ahvaz Jundishapur, University of Medical Sciences, Ahvaz, Iran; UCL Queen Square Institute of Neurology, University College London, London WC1E 6BT, UK; Level 4, RILD Wellcome Wolfson Medical Research Centre, RD&E (Wonford) NHS Foundation Trust, University of Exeter Medical School, Exeter EX2 5DW, UK; Medical Research Council Mitochondrial Biology Unit, University of Cambridge, Cambridge CB2 0XY, UK; Level 4, RILD Wellcome Wolfson Medical Research Centre, RD&E (Wonford) NHS Foundation Trust, University of Exeter Medical School, Exeter EX2 5DW, UK; Peninsula Clinical Genetics Service, Royal Devon and Exeter Hospital (Heavitree), Exeter EX1 2ED, UK

**Keywords:** TMEM63C, hereditary spastic paraplegia/HSP, mitochondria, endoplasmic reticulum/ER, mitochondria-ER contact sites/MERCs

## Abstract

The hereditary spastic paraplegias (HSP) are among the most genetically diverse of all Mendelian disorders. They comprise a large group of neurodegenerative diseases that may be divided into ‘pure HSP’ in forms of the disease primarily entailing progressive lower-limb weakness and spasticity, and ‘complex HSP’ when these features are accompanied by other neurological (or non-neurological) clinical signs. Here, we identified biallelic variants in the transmembrane protein 63C (*TMEM63C*) gene, encoding a predicted osmosensitive calcium-permeable cation channel, in individuals with hereditary spastic paraplegias associated with mild intellectual disability in some, but not all cases. Biochemical and microscopy analyses revealed that TMEM63C is an endoplasmic reticulum-localized protein, which is particularly enriched at mitochondria–endoplasmic reticulum contact sites. Functional *in cellula* studies indicate a role for TMEM63C in regulating both endoplasmic reticulum and mitochondrial morphologies. Together, these findings identify autosomal recessive *TMEM63C* variants as a cause of pure and complex HSP and add to the growing evidence of a fundamental pathomolecular role of perturbed mitochondrial-endoplasmic reticulum dynamics in motor neurone degenerative diseases.

## Teaser

Al-Salmi *et al.* show that *TMEM63C* variants cause HSP with/without intellectual disability. Consistent with lipidomic imbalance being a common feature of HSP, they show *in cellulo* that TMEM63C is enriched at mitochondrial-ER contact sites and that *TMEM63C* knockout causes mitochondrial morphology defects.

## Introduction

Hereditary spastic paraplegia (HSP) was first described by Strumpell and Lorrain in the late 19th century and was initially considered to be a small group of Mendelian disorders. However, subsequent advancements in our understanding of the genetic architecture of HSP have led to it being recognized as one of the most genetically (>80 causative genes) and clinically heterogeneous of inherited diseases. HSP is characterized clinically by lower-limb spasticity and weakness, and pathologically by the retrograde degeneration of motor neurons.^1,2^ HSP may be subdivided into pure and complex forms, depending on whether other system involvement or neurological features accompany the cardinal clinical sign of progressive lower-limb spastic weakness. Autosomal recessive forms account for an estimated 25–30% of HSP patients, typically involving complex forms of HSP with additional clinical features including impaired vision and hearing, cognitive impairment, seizures and peripheral neuropathy.^1^ At present, there is no cure for the condition and treatments are largely symptomatic, involving the use of antispasmodic agents such as baclofen, progabide and dalfampridine.^3^ Increasing understanding of the biological basis of HSP is supporting the development of new treatments, which have the potential to be personalized depending on the underlying genetic cause.^3^

The significant molecular heterogeneity of HSP is indicative of a complex pathomolecular aetiology, which remains poorly understood with genes associated with the condition being implicated in a wide array of cellular processes. These include protection against oxidative stress, DNA repair, metabolism of neuroprotective steroids, myelin sheath stabilization, axonal growth and subcellular transport,^4^ all proposed to lead to axonal failure and progressive lower-limb spasticity characteristic of the condition.^1,3^ Recently, increasing genetic and molecular evidence suggests a potential central role for aberrant lipid metabolic processes in particular involving endoplasmic reticulum (ER), mitochondria and other organelles.^2^ Many of the genes associated with motor neuron degenerative diseases, including HSP, have been linked in molecular studies with lipid metabolic pathways, in particular involving molecular flux between the ER and mitochondria. ER and mitochondrial compartments connect via mitochondria-ER contact sites (MERCs),^2^ which are distinct structural domains characterized by the close apposition of both ER and mitochondrial membranes, and establish a molecular platform crucial for signalling and metabolite flux between both organelles in order to maintain organelle and cellular homeostasis.^5–7^ The ER is the main site of phospholipid biosynthesis and provides lipid precursors to other membranes, including mitochondria.^8,9^ Phospholipid transport from the ER to mitochondria via MERCs enables the synthesis of essential mitochondrial phospholipids including cardiolipin, phosphatidylserine and phosphatidylethanolamine.^10^ These molecular pathways provide the essential building blocks of biological membranes, and alterations in genes encoding the proteins regulating these pathways have been linked with HSP.^2,10–12^ Thus, maintaining MERCs integrity is increasingly recognized as being critical for phospholipid metabolism and also cellular homeostasis more widely,^13^ and gene alterations leading to impaired MERCs function probably form a molecular theme common to many neurological disorders.^2,14^

The osmosensitive calcium (OSCA)/transmembrane protein 63 (TMEM63) protein family members entail a newly identified family of mechanical ion channels activated by membrane tension, which are conserved across eukaryotes.^15^ The family comprises three members: *TMEM63A*, *TMEM63B* and *TMEM63C*, the function of which has not been extensively explored in mammals. Zhao *et al*.^16^ identified a possible role of *TMEM63* proteins as osmoreceptor transduction channels, and found that expression of all three members was required in cell transfection studies for channel activity. Studies on *TMEM63C* plant orthologues (AtCSC1 and OSCA1) indicate that it may indeed function as a Ca^2+^ permeable cation channel that is activated by hyperosmotic stress.^15,16^ While previous studies of TMEM63C are restricted to its plant orthologues, maintenance of the ionic and osmotic composition and volume of fluids is crucial for the normal functioning of the brain,^17^ of clear relevance should the mammalian (and human) *TMEM63C* orthologues possess similar functionalities. Interestingly, studying the structure of ER-localized OSCA1.2, a plant orthologue of *TMEM63C*, revealed striking topological similarities with the transmembrane protein *TMEM16.*^18,19^ Dysfunction of different TMEM16 proteins (also known as anoctamins) is associated with several neurologic disorders, including muscle disease, cerebellar ataxia and dystonia.^20^ Importantly, one member of this family, TMEM16K, has been proposed to regulate endosomal function at ER-endosome contact sites. Furthermore, the yeast TMEM16 orthologue, Ist2p, is a tether protein connecting the ER and the plasma membrane, identifying a role of some TMEM16 family members at membrane contact sites.^21,22^ Here we present genetic, clinical and molecular data that identify biallelic variants in *TMEM63C* probably leading to loss of function, as a cause of both pure and complex HSP. Our molecular studies determine that TMEM63C is an ER-localized protein enriched at MERCs, and that *TMEM63C* knockdown is associated with mitochondrial and ER morphological defects, revealing a previously unidentified role of TMEM63C in mediating organelle homeostasis.

## Materials and methods

### Clinical studies

All research was performed with informed consent from the study participants or their legal guardians, and according to institutional and international guidelines for studies with human subjects and materials (approved protocols; RC/SCI/BIOL/10/01 and EE/97.24.3 17654/scu.ac, ir). Affected individuals were investigated according to routine clinical standards for the diagnosis of neurological disease.

### Genetic studies

DNA was extracted from blood using standard techniques. Single nucleotide polymorphism (SNP) genotyping was carried out on DNA from both affected individuals from Family 1 (IV:2, IV:8), using Illumina Human CytoSNP-12v2.1 arrays.

In all three families, whole exome sequencing (WES) was undertaken to identify the cause of disease. Genomic variants were filtered based on call quality, impact on gene function, allele frequency in the population databases and segregation with disease. Variants were then assessed for clinical correlation with the affected individual(s) phenotype. In Family 1, WES was performed in Exeter on DNA from affected Individual IV:2 (NextSeq500; Illumina, San Diego, CA, USA) and involved Agilent Sureselect Whole Exome v.6 (Agilent Technologies, Santa Clara, CA, USA) targeting, read alignment (BWA-MEM v.0.7.17), mate-pairs fixed and duplicates removed (Picard v.2.15), InDel realignment and base quality recalibration (GATK v.3.7.0), single nucleotide variant and InDel detection (GATK HaplotypeCaller), variant annotation [Alamut batch v.1.10 and SnpEff (http://snpeff.sourceforge.net/SnpEff_manual.html)] and read depth assessment (equivalent to GATK DepthOfCoverage). Copy number variants (CNVs) were detected using SavvyCNV. Variants with <5 reads and/or a frequency >0.1% in public genome databases including the Genome Aggregation Database (gnomAD v2.1.1) and the 1000 Genomes Project were excluded. Homozygous and compound heterozygous variants that were exonic and non-synonymous, synonymous with predicted splicing impact or intronic at ±6 nucleotides from splice site were evaluated and cross referenced against the SNP data. In Family 2, WES (VI:2) and genome-wide SNP genotyping (affected Individuals: V:I, V:II, VI:I, VI:II) were performed in parallel as previously described.^23^ Variants were prioritized by call quality and frequency <0.1% (gnomAD v.2.1.1) and cross referenced against the SNP data. The variant filtering steps followed in Family 2 were otherwise similar to those described previously. In Family 3, duo WES was performed on DNA from the proband (II:1) and her mother (NextSeq 500 Sequencing System, Illumina, San Diego, USA), with a 2 × 150 bp high output sequencing kit after 12-plex enrichment with SeqCap EZ MedExome kit (Roche). Sequence quality was assessed with FastQC v.0.11.5 and reads mapped and indexed [BWA-MEM (v.0.7.13 and Samtools v.1.4.1)], duplicates flagged (Sambamba v.0.6.6) and coverage calculated (Picard tools v.2.10.10). SNVs/InDels calling was performed using GATK 3.7 Haplotype Caller and CNV detection with CNVKit and Excavator2, variants were annotated using Annovar. The variant filtering steps followed were similar to those described for Family 1.

Unique primers were designed and used for dideoxy sequencing confirmation and cosegregation of genomic variants.

### Molecular and cellular studies

#### Cell culture and transfections

HeLa and SH-SY5Y cells were obtained from American Type Culture Collection and NSC-34 cells from Cedarlane Laboratories (via tebu-bio). HeLa and NSC-34 cells were cultured in Dulbecco’s modified Eagle medium (DMEM) (GIBCO) and SH-SY5Y cells in DMEM/F12 medium (GIBCO), all supplemented with high-glucose (5 g.l^−1^), 10% foetal bovine serum (FBS), 2 mM l-glutamine, 1% non-essential amino acids and 100 units/ml penicillin/streptomycin (all from GIBCO). Cells were maintained at 37°C with 5% CO_2_. NSC-34 cells were differentiated into neurons by maintaining them in DMEM/F12 medium supplemented with 1% FBS, 1% non-essential amino acids and 10 μM retinoic acid (Scientific Laboratory Supplies) for 14 days.^24^

Cells at 80% of confluence were transfected for 24 h using Fugene HD transfection reagent (Promega) following the manufacturers’ instructions. For small interference (si) RNA experiments, cells were reverse transfected using Lipofectamine RNAimax (Invitrogen) with 20 nM siRNA for 3 days, following the manufacturer’s instructions. Media was changed 6 and 24 h after plasmid and siRNA transfections, respectively. NSC-34 cells were silenced after 8 days of differentiation for 6 days with 20 nM siRNA, by performing two consecutive rounds of reverse transfection with Lipofectamine RNAimax (one every 3 days). Cells were tested for mycoplasma contamination using the Lookout Mycoplasma PCR detection kit (Sigma).

#### siRNA oligonucleotides and plasmids

The following siRNAs were obtained from Dharmacon: non-targeting siRNA (siNT) (ONTARGETplus SMARTpool D-0001810-10-20), siRNA targeting human *TMEM63C* (siTMEM63C) (ONTARGETplus SMARTpool L-021981-02-0005) and siRNA targeting mouse *TMEM63C* (siTMEM63C) (ONTARGETplus SMARTpool L-055603-01-0005). IDT codon optimization tool was used to design a Gblock containing the full sequence of TMEM63C including a Flag sequence at the C-terminal region and extensions to the 5′ and 3′ ends to enable In-Fusion cloning technology (Takara). The insert was introduced into a pcDNA3.1(+) plasmid at the KpnI and XbaI restriction sites. pcDNA3.1(+) backbone (empty vector) was used as control.

#### Antibodies

The following primary antibodies and dilutions were used for immunofluorescence studies: mouse anti-Flag (Sigma-Aldrich, F3165) (1:1000), rabbit anti-TOM20 (Santa-Cruz, sc11415) (1:1000), rabbit anti-PMP70 (Sigma-Aldrich, SAB4200181) (1:1000), mouse anti-p230 (BD Biosciences, 611281) (1:1000), rabbit anti-Rab7 (Abcam, ab137029) (1:1000), rat anti-Calnexin (Biolegend, 699401) (1:1000) and mouse anti-Neurofilament H (Biolegend, 835801). Donkey anti-mouse, Goat anti-mouse IgG1, Goat anti-mouse IgG2a, Goat anti-rabbit and Goat anti-rat, Alexa Fluor 488, 568 or 647 were used as secondary antibodies (1:1000) (Invitrogen).

The following primary antibodies and dilutions were used for immunoblot analysis: mouse anti-Flag (Sigma-Aldrich, F3165) (1:1000), mouse anti-VDAC1 (Abcam, ab14734) (1:1000), rabbit anti-TMEM63C (Abcam, ab203486) (1:500), rabbit anti-Pex14 (Proteintech, 10594-1-AP) (1:1000), rabbit anti-Calnexin (Proteintech, 10427-1-AP) (1:1000), mouse anti-Tubulin (Santa-Cruz, sc23948) (1:1000), rabbit anti-VAPB (Atlas, HPA013144) (1:500) and mouse anti-ACSL4 (Santa-Cruz, sc-365230) (1:1000). Horseradish peroxidase-conjugated anti-rabbit and anti-mouse IgG (GE Healthcare) were used as secondary antibodies (1:3000).

#### SDS–PAGE and immunoblotting

Cells were lysed in RIPA buffer (20 mM Tris pH 8.0, 150 mM NaCl, 0.1% SDS, 1% deoxycholic acid, 1% NP-40 and complete protease inhibitor cocktail). Protein concentration of the samples was measured using Bradford protein assay (BioRad) calibrated using a bovine serum albumin standard curve. Proteins were resolved by sodium dodecyl sulphate–polyacrylamide gel electrophoresis (SDS–PAGE) and then transferred to nitrocellulose membranes (0.2 μm pore size, GE Healthcare). Membranes were blocked with 5% of skimmed milk in PBS for 1 h at room temperature to block non-specific epitopes. Membranes were then incubated with the appropriate primary antibodies diluted in 2% milk-PBS-0.05% Tween-20 at 4°C overnight. Membranes were washed in 0.05% Tween-20 in PBS three times for 15 min and incubated with appropriate secondary antibodies (1/3000 in 2% Milk-0.05% Tween-20 in PBS) and treated with Western Lightning Plus ECL (Perkin Elmer). Chemiluminescent signal was captured using films (PROTEC®) or on a digital ECL machine (Amersham). Uncropped immunoblots for all the experiments presented in the figures are displayed in Supplementary Fig. 4.

#### Immunofluorescence

Immunofluorescence experiments were performed as previously described.^25^ Briefly, cells were fixed in 5% paraformaldehyde (PFA) in PBS at 37°C for 15 min, then washed three times with PBS, followed by incubation with 50 mM ammonium chloride in PBS to quench the unspecific fluorescence signal from aldehyde groups. Cells were washed again three times with PBS and permeabilized in 0.1% Triton X-100 in PBS for 10 min. Then, cells were blocked with 10% FBS in PBS, followed by incubation with the appropriate primary antibodies in 5% FBS in PBS, for 2 h at room temperature. After three washes with 5% FBS in PBS, cells were incubated with specific secondary antibodies (1:1000) for 1 h at room temperature. After three washes in PBS, coverslips were mounted onto slides using Dako fluorescence mounting medium (Dako) (confocal imaging) or ProLong^TM^ Diamond Antifade (Thermo) (super-resolution imaging). For nuclear staining, coverslips were mounted using ProLong™ Gold Antifade Mounting with DAPI (Thermo).

#### Confocal and N-structured illumination microscopy image acquisition

For confocal imaging, fixed and stained cells were visualized and acquired using a ×100 objective lens (numerical aperture 1.4) on a Nikon Eclipse TiE inverted microscope with appropriate lasers using an Andor Dragonfly 500 spinning disc system, equipped with a Zyla 4.2 PLUS sCMOS camera (Andor), coupled with Fusion software (Andor). For TMEM63C co-localization, mitochondrial and ER morphology analysis, seven stacks of 0.2 μm each were acquired using the ×100 objective and then compiled by ‘max projection’ using the Fiji software. Mitochondrial morphology was analysed and presented as intermediate, elongated or fragmented, as described previously.^26^ Mitochondria were classified as ‘fragmented’ when most of the mitochondria of the cell were short and spherical, ‘elongated’ when most of the mitochondria of the cell presented highly elongated mitochondria with <10 free ends, and ‘intermediate’ when most of the mitochondria of the cell were tubular, neither connected or spherical. For N-Structure Illumination Microscopy super-resolution imaging, fixed samples were observed under a Nikon Eclipse Ti-E microscope equipped with an Andor iXon camera coupled with a Nikon N-SIM attachment. Seven stacks of 0.2 μm each were acquired using the ×100 objective coupled with Nis-Elements AR 5.21.03 software.

**Figure 3:**
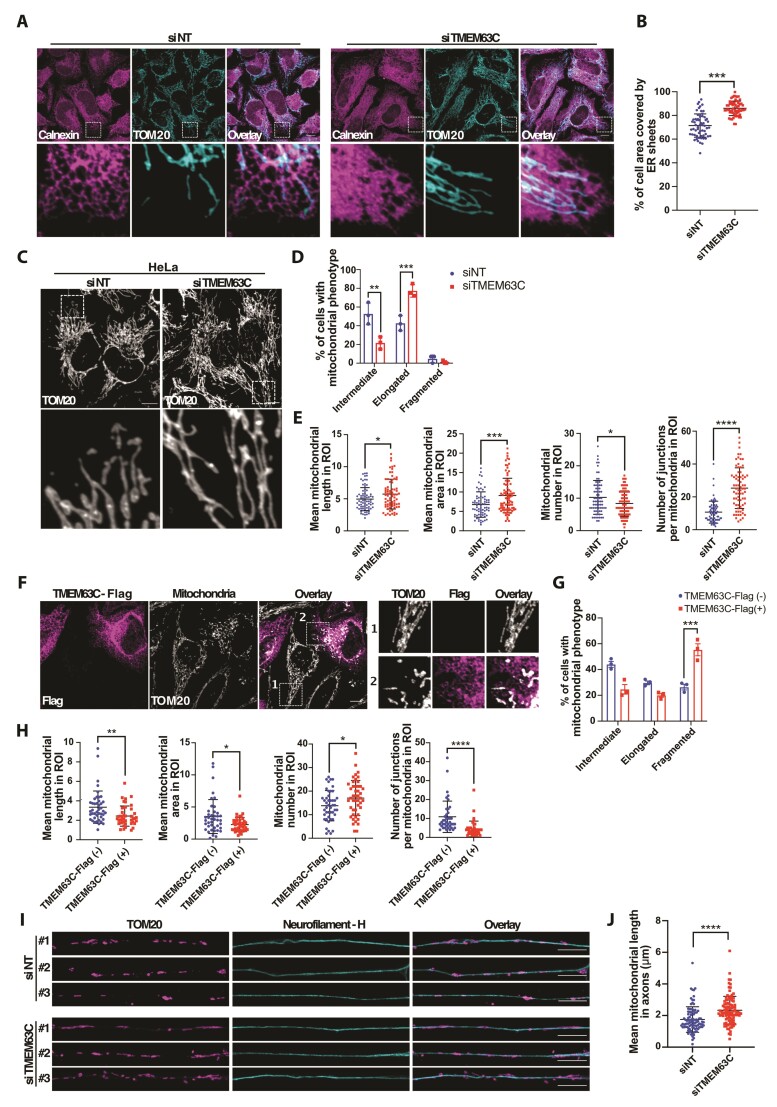
**TMEM63C silencing alters both ER and mitochondrial morphologies.** (**A**) Representative confocal images of ER morphology of control (siNT) and *TMEM63C* (siTMEM63C) silenced HeLa cells. ER and mitochondria were labelled with anti-Calnexin and anti-TOM20 antibodies, respectively. Scale bars = 10 µm. (**B**) Quantification of ER morphology related to **A**. (**C**) Representative confocal images of mitochondrial morphology of control (siNT) and *TMEM63C* (siTMEM63C) silenced HeLa cells. Mitochondria were labelled using an anti-TOM20 antibody. Scale bars = 10 µm. (**D**) Quantification of mitochondrial morphology related to **C**. (**E**) Quantification of different mitochondrial morphology parameters including mean mitochondrial length and area, mitochondrial number and mitochondrial branching measured by mitochondrial junction number, per region of interest of 225 µm^2^, related to **C**. (**F**) Representative confocal images of mitochondrial morphology of TMEM63C-Flag overexpressing HeLa cells, compared to untransfected cells. Flag and mitochondria were labelled with anti-Flag and anti-TOM20 antibodies, respectively. Scale bars = 10 µm. (**G**) Quantification of mitochondrial morphology related to **F**. (**H**) Quantification of different mitochondrial morphology parameters including mean mitochondrial length and area, mitochondrial number and mitochondrial branching measured by mitochondrial junction number, per region of interest of 225 µm^2^, related to **F**. (**I**) Representative confocal images of axonal mitochondria from control (siNT) and *TMEM63C* (siTMEM63C) silenced differentiated motor neuron-like NSC-34 cells. Mitochondria were labelled using an anti-TOM20 antibody and axons were recognized using an anti-Neurofilament H antibody. Scale bars = 10 µm. (**J**) Quantification of axonal mitochondrial length related to **I**. All data are shown as mean ± SD of at least three independent experiments. For **D** and **G**, two-way ANOVA and Tukey’s multiple-comparisons test was used; for **B**, **E**, **H** and **J**, Mann–Whitney U-test (two-tailed) was used. *P < 0.05, **P < 0.01, ***P < 0.001, ****P < 0.0001.

The different mitochondrial parameters (length, area, number and number of junctions) were quantified by randomly selecting regions of interest of 225 μm^2^ at the cell periphery and analysed using MitoMapr (Fiji).^27^ For ER quantification, the total area of the cell and the area covered by ER sheet were manually selected using Fiji tools. Fiji macro (JaCOP) was used to measure Mander’s coefficient of co-localization between the ER and mitochondria. At least 15 cells per condition were analysed; three independent experiments. All the representative images were processed once with the ‘smooth’ function in Fiji. The number of cells analysed in the corresponding figures are: Fig. 3B (siNT = 60 cells, siTMEM63C = 62 cells), Fig. 3D (siNT = 105 cells, siTMEM63C = 93 cells), Fig. 3E (siNT = 76 cells, siTMEM63C = 78 cells), Fig. 3G and H [TMEM63C-Flag (−) = 45 cells, TMEM63C-Flag (+) = 45 cells], Fig. 3J (siNT = 98 axons, siTMEM63C = 101 axons), Supplementary Fig. 3C (siNT = 72 cells, siTMEM63C = 66 cells), Supplementary Fig. 3D (siNT = 57 cells, siTMEM63C = 55 cells) and Supplementary Fig. 3F (siNT = 63 cells, siTMEM63C = 60 cells).

#### Respirometry

Oxygen consumption measurements were performed in intact cells resuspended in culture DMEM medium using an Oroboros Instruments High-Resolution Respirometer.^28^ Approximately 3 × 10^6^ cells were used for each experiment. Basal (ROUTINE) respiration was recorded until the steady state was reached. The non-phosphorylating respiration (LEAK) was measured adding 2.5 μM oligomycin to the chambers to inhibit the ATP synthase and the respiration rates were left to reach the steady state. The uncoupled state or maximal capacity of the electron transfer system was achieved by titrating CCCP in 0.5 μM steps until the respiratory rates did not increase any further. Finally, 2.5 μM antimycin A and 1 μM rotenone were added to inhibit respectively complex III and complex I. *n* = four independent experiments.

#### Mitochondrial-associated membrane isolation

Mitochondrial-associated membrane (MAM) isolation was performed as described previously.^29^ Briefly, HeLa cells were trypsinized, pelleted at 300*g* and washed in 1× PBS, pH 7.4. All the following centrifugations were performed at +4°C. Pellets were resuspended in prechilled mitochondria isolation buffer (MIB) (220 mM mannitol, 70 mM sucrose, 10 mM Tris-KOH pH 7.4, 1 mM EDTA) with protein cocktail inhibitor and homogenized in a manual glass mortar (Kimble). Homogenates were centrifuged at 800*g*. Supernatants were centrifuged at 2300*g* with pellets washed in MIB and collected as the crude mitochondrial fraction. Additional centrifugation at 8000*g* was performed. Supernatants were then centrifuged at 100 000*g* for 60 min and cytosolic (supernatant) and microsomal (pellet) fractions were obtained. The crude mitochondrial fraction was then resuspended in MIB buffer, layered on 30% Percoll solution and centrifuged at 95 000*g* for 65 min. MAM and pure mitochondrial fraction were extracted with 20-gauge needles. MAM and pure mitochondrial fraction were then washed in ice-cold 1× PBS, pH 7.4 and centrifuged at 6300*g* and 10 000*g* for 20 min for MAM and pure mitochondrial fraction, respectively. All fractions were extracted with MIB 1% Triton X-100, normalized for protein content and processed for SDS–PAGE and immunoblotting.

#### Statistical analysis

Errors bars displayed on graphs represent the mean ± SD from at least three independent experiments. Statistical significance was analysed using Mann–Whitney U-test (two-tailed), unpaired *t*-test (two-tailed) or two-way ANOVA test using GraphPad Prism software. *P < 0.05, **P  < 0.01, ***P < 0.001 and ****P < 0.0001 were considered significant.

### Data availability

The authors confirm that the data supporting the findings of this study are available within the article and Supplementary material. Further derived data are available from the corresponding author upon reasonable request.

## Results

### Clinical and genetic studies

**Figure 1:**
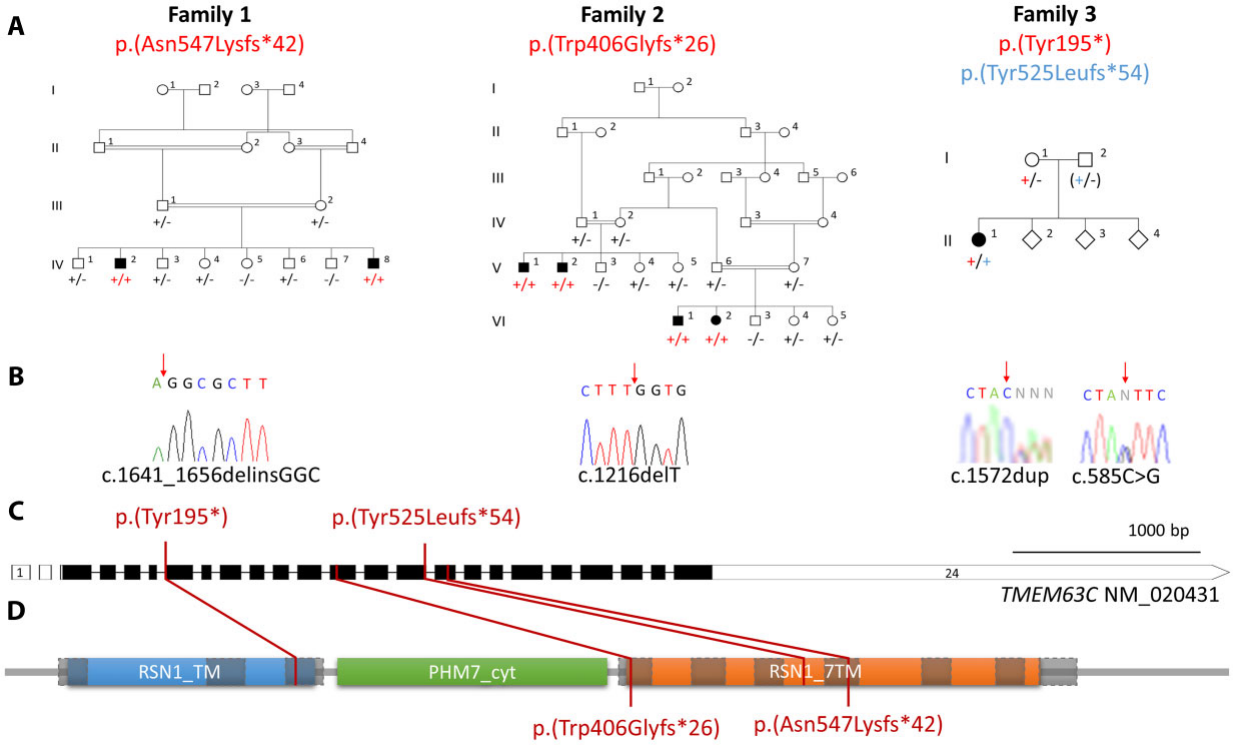
**Family pedigrees and biallelic *TMEM63C* variants identified as a cause of HSP**. (**A**) Family 1: Extended Omani pedigree in which two affected individuals are homozygous for the Chr14(GRCh37):77714729-744delinsGGC; NM_020431.3:c.1641_1656delinsGGC; NM_020431.3:p.(Asn547Lysfs*42) variant. Family 2: Extended Iranian pedigree including two nuclear families with a total of four affected individuals homozygous for the Chr14(GRCh37):g.77709274delT, NM_020431.3, c.1216delT, p.(Trp406Glyfs*26) variant. Family 3: North African pedigree with one affected individual compound heterozygous for both p.(Tyr195*) and p.(Tyr525Leufs*54) variants. Cosegregation confirmed in other family members as indicated, in each case ‘+’ indicating variant allele and ‘−’ indicating wild-type allele (paternal genotype inferred in Family 3, indicated by genotyping shown in parentheses). (**B**) Dideoxy sequence chromatogram of an affected individual from each pedigree showing variant alleles. (**C**) Schematic diagram of the TMEM63C gene showing intron/exon genomic organization and the location of *TMEM63C* gene variants. (**D**) Schematic diagram of the TMEM63C polypeptide showing location of the variants identified with regard to protein domain architecture. Protein domains: RSN1_TM = late exocytosis, associated with Golgi transport, PHM7_cyt = cytosolic domain of 10TM putative phosphate transporter, RSN1_7TM = RSN1_7 calcium-dependent channel, seven transmembrane region.

We initially investigated the cause of disease in two male Omani siblings (Family 1-IV:2 and IV:8) (Fig. 1) affected by HSP associated with mild intellectual impairment. The older male (IV:2) was late to walk and presented at 19 months of age with lower-limb spasticity and weakness. Although his phenotype was initially thought to be pure HSP, he was later noted to be microcephalic (−3.78 SD) and to be cognitively impaired [Stanford Binet Intelligence Scale (IQ) score of 61 (attained mental age of 7 years 6 months at a chronological age of 11 years 4 months)]. Upper limb reflexes, motor function and sensation were all unaffected and there was no clinical evidence of bulbar involvement. Lower-limb nerve conduction studies (NCS) and MRI were performed and were unremarkable. The younger brother (IV:8), followed a very similar clinical course. The earliest sign of neurological impairment was delayed motor milestones (crawled at 12 months, independent walking 22 months) and toe walking. At age 3 years, he had progressive lower-limb spastic weakness and mild global developmental delay with limited vocabulary and language processing skills. To identify the genetic cause of HSP, genome-wide SNP mapping was undertaken on DNA from both affected brothers (IV:2 and IV:8) in parallel with WES in IV:2, assuming homozygosity for a founder mutation was most probably responsible, although also considering other possible genetic causes. As expected in this family structure, autozygosity mapping identified 14 genomic regions >1 Mb common to both affected siblings, among the largest being a ∼10 Mb region on chromosome 14q24 (Supplementary Fig. 1). WES identified a homozygous [Chr14 (GRCh37):77,714,729-744delinsGGC; NM_020431.3:c.1641_1656delinsGGC; NM_020431.3: p.(Asn547Lysfs*42)] variant in the transmembrane protein 63C (*TMEM63C*) gene as the most likely cause of disease, located within the shared chromosome 14q24 genomic region. The variant, which is not listed in gnomAD v.2.1.1 or v.3.1.1 nor in our in-house ancestry-matched Omani database of 1562 whole exomes, is predicted to cause a frameshift beginning at codon asparagine 547 leading to a premature stop codon 42 amino acids subsequently [p.(Asn547Lysfs*42)]. Other variants that could not be excluded in this family are listed in Supplementary Table 1.

Family 2, an extended Iranian family, comprises two nuclear families each with two siblings diagnosed with HSP (Family 2-VI:1, VI:2, V:1, V:2) (Fig. 1). All four affected individuals presented with delayed motor development and displayed progressive lower-limb spasticity with associated hyper-reflexia, upgoing plantar responses, clonus and muscle weakness. This resulted in a narrow-based spastic gait and excessive lordosis in Individual VI:1 (Supplementary Video 1). There was no evidence of upper limb involvement on examination of VI:1 and VI:2. Ophthalmological findings included strabismus (VI:2) and horizontal nystagmus (V:1). Both affected Individuals VI:1 and V:1 have a mild stutter. Siblings VI:1 and VI:2 have normal intellect, whereas both V:1 and V:2 displayed signs of mild intellectual impairment. MRI, NCS and electromyography were undertaken as part of VI:1 and VI:2’s clinical investigations and were unremarkable. Exome sequencing undertaken on DNA from VI:2 identified a homozygous [Chr14(GRCh37): g.77709274delT, NM_020431.3, c.1216delT, p.(Trp406Glyfs*26)] variant in *TMEM63C*, predicted to cause a frameshift beginning at codon 406 and leading to a premature stop codon 26 amino acids downstream, as the only candidate causative mutation after correlation with the genome-wide SNP mapping data. The variant is not present in either gnomAD v.2.1.1 or v.3.1.1 and is located in a 9.2 Mb region of homozygosity on chromosome 14q24.3 (Supplementary Fig. 1).

Following these findings in Families 1 and 2, we next explored GeneMatcher and identified a north African family (Family 3) (Fig. 1) with a single female (Family 3-II:1) affected with HSP diagnosed in early childhood. At age 20 years she has mild intellectual impairment and remains ambulatory. WES identified compound heterozygous Chr14(GRCh37): g.77703009C > G, NM_020431.3:c.585C > G, p.(Tyr195*) and Chr14(GRCh37) and g.77712988dup, NM_020431.3:c.1572dup, p.(Tyr525Leufs*54), probable loss of function variants in *TMEM63C*, both absent in gnomAD v.2.1.1 and 3.1.1.

In all three pedigrees the TMEM63C variants were validated by dideoxy sequencing and found to cosegregate in all available family members as appropriate for autosomal recessive inheritance (Fig. 1) (see Table 1, Supplementary Fig. 1 and Supplementary Table 1 for detailed clinical summaries, genome-wide SNP mapping data and WES variant lists).

**Table 1:** Clinical findings in affected individuals with biallelic *TMEM63C* variants

Family	Family 1		Family 2				Family 3
Pedigree reference	IV:2	IV:8	VI:1	VI:2	V:1	V:2	II:1
Genotype	p.(Asn547Lysfs*42)/p.(Asn547Lysfs*42)		p.(Trp406Glyfs*26)/p.(Trp406Glyfs*26)				p.(Tyr195*)/p.(Tyr525Leufs*54)
Gender	Male	Male	Male	Female	Male	Male	Female
Ethnicity	Omani	Omani	Iranian	Iranian	Iranian	Iranian	North African
Age at evaluation (years)	17	3	8	26	30	32	15
Age of symptom onset	19 months	18 months	Infancy	Infancy	Infancy	Infancy	6 months
**Growth parameters**							
Height cm (SDS)	165 (−1.52)	90 (−1.49)	118 (−1.8)	156 (−1.3)	NK	NK	162 (−0.03)
Weight kg (SDS)	47.1 (−2.29)	11.5 (−2.28)	21 (−1.52)	55 (−0.42)	NK	NK	67 (1.45)
Head circumference cm (SDS)	50.5 (−3.78)	50.5 (−0.69)	51 (−1.83)	58 (1.79)	NK	NK	54.5 (−0.45)
**Development**							
Intellectual disability	Mild, IQ 62	Mild	−	−	Mild	Mild	Mild
Gross motor	Delayed walking	Crawled at 12 months, walked at 22 months	Walked at 22 months	Walked at 22 months	Delayed walking	Delayed walking	Walked at 20 months
Speech	Normal	Limited vocabulary, only 2 words until 24 months	Stuttering	Normal	Stuttering	Normal	Normal
Vision	Normal	Normal	Normal	Strabismus	Nystagmus	Normal	Normal
Hearing	Normal	Normal	Normal	Normal	Normal	Normal	Normal
Developmental regression	−	−	−	−	−	−	−
**Neurology**							
Lower limb							
Spasticity	+	+	+	+	+	+	+
Hyper-reflexia	+	+	+	+	+	+	+
Extensor plantars	+	+	+	+	NK	NK	+
Upper limb							
Spasticity	−	−	−	−	NK	NK	−
Hyper-reflexia	−	−	−	−	NK	NK	−
Cerebellar signs	−	−	−	−	NK	NK	−
Dystonia	−	−	−	−	NK	NK	−
Dysarthria	−	−	−	−	−	−	−
Gait	Spastic	Spastic	Spastic	Spastic	Spastic	Spastic	Spastic
Other clinical findings			Lumbar lordosis	Lumbar lordosis	Lumbar lordosis	Lumbar lordosis	Hypertonic urinary bladder disturbance
**Investigations**							
MRI brain	NAD	NP	NAD	NAD	NP	NP	NP
Other investigations	NCS-NAD VEP-NAD BAEP-NAD		NCS-NAD EMG-NAD	NCS-NAD EMG-NAD			

NP = not performed; SDS = standard deviation score; (+) = indicates presence of a feature in an affected individual; (−) = indicates absence of a feature in an affected individual; NK = not known; NAD = no abnormality detected; NCS = nerve conduction studies; VEP = visual evoked potential; BAEP = brainstem auditory evoked potential. Height, weight and standard deviation scores were calculated using a Microsoft Excel add-in to access growth references based on the LMS method using UK 1990 reference population.^30^

### Molecular studies

**Figure 2:**
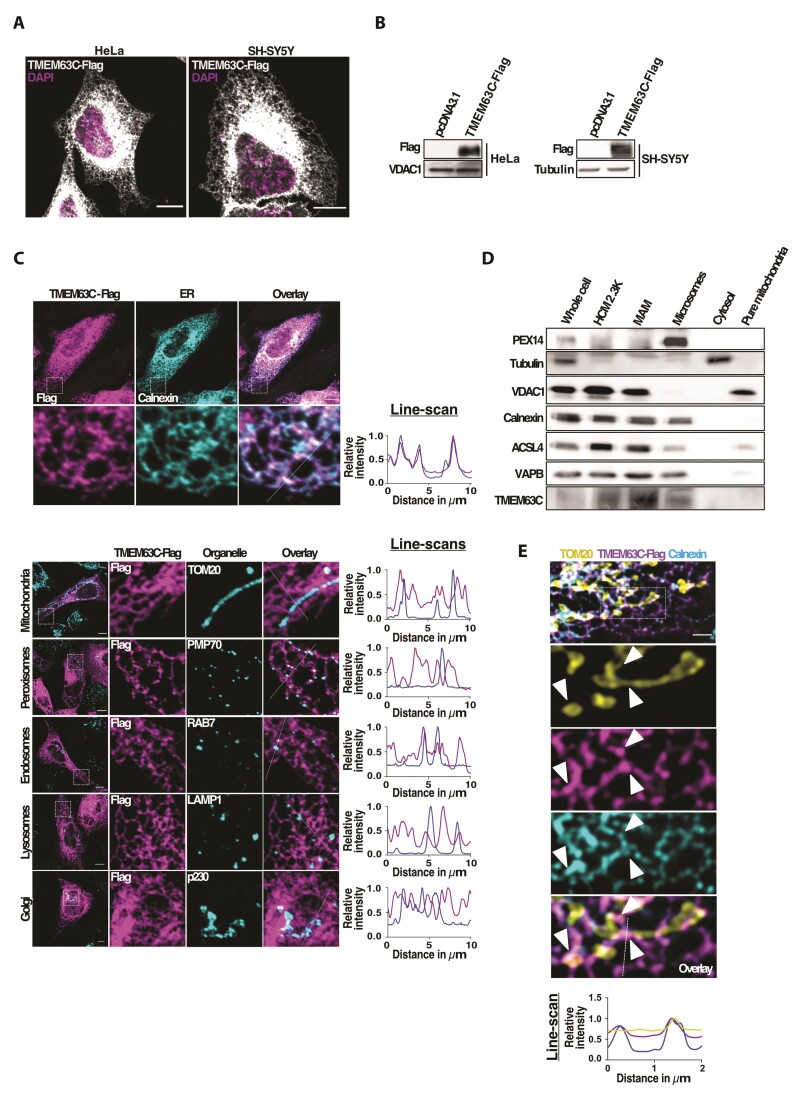
**TMEM63C distributes along the ER network and accumulates at MERCs.** (**A**) Representative confocal images of HeLa and SH-SY5Y cells transfected with TMEM63C-Flag and labelled with an anti-Flag antibody. Nucleus were labelled using DAPI. Scale bars = 10 µm. (**B**) Immunoblot analysis showing the efficiency of TMEM63C-Flag expression at 24 h in both HeLa and SH-SY5Y cells. VDAC1 and Tubulin were used as loading controls. (**C**) Representative confocal images of HeLa cells transfected with TMEM63C-Flag showing TMEM63C-Flag co-localization with the ER marker, Calnexin (*top*), compared to other organelle markers (*bottom*). Mitochondria, peroxisomes, endosomes, lysosomes and Golgi apparatus were labelled using anti-TOM20, anti-PMP70, anti-Rab7, anti-LAMP1 and anti-p230 antibodies, respectively. Flag was labelled using an anti-Flag antibody. On the *right*, 10 µm line-scan analyses of relative fluorescence intensity from the dashed line are shown. Scale bars = 10 µm. (**D**) TMEM63C localization analysis by subcellular fractionation from HeLa cells. Total cell lysates (whole cell) were fractionated into cytosolic (cytosol), heavy crude mitochondria (HCM 2.3K), purified mitochondria (pure mitochondria), MAMs and microsomal (microsomes) fractions. The following proteins were used as compartment markers: VDAC1 for outer mitochondrial membrane and MAM, calnexin for ER and MAM, ASCL4 and VAPB for MAM, PEX14 for peroxisomes and Tubulin for cytosol. (**E**) Representative N-SIM super-resolution microscopy image of HeLa cells expressing TMEM63C-Flag, showing TMEM63C-Flag *foci* accumulation at MERCs (arrows). Flag, mitochondria and ER were labelled with anti-Flag, anti-TOM20 and anti-Calnexin antibodies, respectively. On the *right*, 2 µm line-scan analyses of relative fluorescence intensity from the dashed line are shown. Scale bars = 2 µm.

To gain an initial insight into the molecular function, a characterization of TMEM63C was performed using two different mammalian cell lines: the HeLa cell line derived from cervical cancer cells and the neuroblastoma SH-SY5Y cells, which both represent well established models for molecular and cellular studies. First, we aimed to elucidate the cellular distribution of TMEM63C. Since commercial antibodies did not work for immunofluorescence staining, we developed a codon-optimized TMEM63C construct tagged with a C-terminal FLAG epitope to analyse the intracellular localization of TMEM63C. While TMEM63 family members have been proposed to be localized to the plasma membrane,^15,31^ confocal microscopy analysis of TMEM63C-FLAG overexpressing HeLa cells revealed expression at a specific intracellular location reminiscent of an ER protein (Fig. 2A and B). Precise subcellular localization of TMEM63C was then performed by confocal microscopy in TMEM63C-FLAG overexpressing HeLa cells counterstained for other cellular organelles. Co-localization analysis showed that TMEM63C-FLAG specifically and solely localized to the ER, and not to mitochondria, peroxisomes, lysosomes, endosomes, nor the Golgi apparatus (Fig. 2C). The ER is not only the major cellular storage for lipids and Ca^2+^, but also represents the cellular organelle that establishes the largest number of physical contacts with other organelles at membrane contact sites.^5^ Among them, MERCs have been the best characterized to date.^5^ MERCs or MAMs represent functional signalling platforms where specialized ER subdomains in close contact with the mitochondria control different physiological functions like the trafficking of lipids and Ca^2+^ required to maintain cellular homeostasis.^32^ Interestingly, it has been recently highlighted that MERCs play a central role in multiple neurodegenerative diseases, including HSP.^33,34^ Thus, we hypothesized that TMEM63C may localize at this interface. Subcellular fractionation experiments were performed by differential centrifugation to isolate intracellular compartments including heavy membranes, microsomes (containing ER), pure mitochondria and MAM fractions. Notably, immunoblot analysis of these isolated fractions confirmed the presence of TMEM63C in microsomes, but also identified an enrichment of TMEM63C in the isolated MAM fraction, indicating a localization of the protein at this interface (Fig. 2D). To confirm this, we performed super-resolution N-structured illumination microscopy (N-SIM), which allow us to observe not only the TMEM63C ER distribution (revealed by its co-localization with the ER marker calnexin), but also the presence of TMEM63C-enriched ER subdomains in close contact with the mitochondrial marker TOM20 (Fig. 2E and Supplementary Fig. 2). Thus, these data indicate that TMEM63C is an ER-localized protein, which is particularly enriched at MERCs.

Then, we evaluated how the absence of TMEM63C affects organelle homeostasis. To this purpose, we first confirmed the endogenous expression and the efficiency of the siRNA-mediated downregulation of *TMEM63C* gene by immunoblot analysis in the different cell lines used in this study (HeLa, SH-SY5Y and NSC-34 cells) (Supplementary Fig. 3A). To characterize the physiological relevance of TMEM63C at MERCs, potential abnormalities in mitochondrial and ER dynamics were monitored in TMEM63C-deficient cells. The ER membranous compartment consists of a nuclear envelope and a dynamic network of tubules. ER tubules are densely packed in the juxtanuclear region forming ER sheets, required for protein synthesis.^35^ At the periphery of the cell, the ER tubular conformation is less packed allowing the generation of curved membranes, which generate ER-derived vesicles.^35^ Therefore, maintaining a proper ratio between ER sheets and tubules is essential for cellular viability. Indeed, multiple ER-shaping proteins have been involved in HSP and the loss of HSP-associated molecules leads to the expansion of ER sheets at the cell periphery.^36^ Interestingly, silencing of *TMEM63C* in HeLa cells disrupted the balance between sheets and tubules by increasing ER-sheet area (Fig. 3A and B), suggesting that ER defects may comprise a key pathomolecular aspect of TMEM63C-associated neurological disease.

A number of ER-shaping or MERCs-localized proteins have been shown to control mitochondrial functions, in particular regulating mitochondrial morphology.^37–39^ Mitochondria are dynamic organelles constantly adapting their shape depending on the cellular metabolic state by undergoing continuous cycles of fission and fusion events.^40^ The essential role of MERCs in this process is well described, and altered mitochondrial shape has been widely reported in multiple neurodegenerative conditions including HSP.^41–45^ Given the MERCs localization of TMEM63C, we thus monitored mitochondrial morphology in cells silenced for *TMEM63C* in both HeLa (Fig. 3C–E) and SH-SY5Y (Supplementary Fig. 3B–D) cells. This led to a significant remodelling of the mitochondrial network characterized by an increased number of cells harbouring elongated and interconnected mitochondria (Fig. 3C and D and Supplementary Fig. 3B and C), an increase of the organelle area and size, and a decrease in mitochondrial number in the region of interest (Fig. 3E and Supplementary Fig. 3D). Moreover, loss of TMEM63C also induced mitochondrial branching, leading to a highly interconnected network and an increase of mitochondrial intersections or junctions (Fig. 3E and Supplementary Fig. 3D), reinforcing the elongated mitochondrial shape observed in TMEM63C-depleted cells. In addition, TMEM63C gain of function analysis revealed opposite effects on mitochondrial morphology. Indeed, TMEM63C overexpression leads to mitochondrial fragmentation characterized by an increased number of cells harbouring small-round shaped mitochondria, as well as a decrease of organelle area and size, accompanied by an increase in mitochondrial number in the region of interest (Fig. 3F–H). This further supports an important role of TMEM63C in regulating mitochondrial morphology at MERCs. However, despite the altered mitochondrial morphology, mitochondrial respiration measured by oxygraphy was not impaired in the absence of TMEM63C, although maximal respiration was slightly decreased in *TMEM63C*-silenced SH-SY5Y cells after 6 days of inhibition (Supplementary Fig. 3E). Finally, we recapitulated the mitochondrial morphology phenotype in NSC-34 cells, a motor neuron-like cellular model usually used to investigate the physiopathological mechanisms of motor neuron disease and therefore more relevant to HSP.^46^ To this end, we differentiated and matured NSC-34 cells into motor neuron-like cells with several morphological properties resembling those of motor neurons. As shown in Fig. 3I, axons positive for the neuronal marker Neurofilament H were selected and axonal mitochondrial morphology was analysed in the presence or absence of TMEM63C. Quantification of the mitochondrial length (Fig. 3I and J) revealed an increased mitochondrial size in the axons of TMEM63C-deficient motor neuron-like cells, further confirming a potential role of the protein in regulating mitochondrial morphology in the nervous system.

Together, these data indicate that TMEM63C localizes to ER and MERCs, where it is important to maintain ER and mitochondrial morphologies and dynamics. It should be noted that microscopy co-localization analysis using Mander’s coefficient showed that loss of TMEM63C did not lead to a decrease but rather a slight increase of MERCs, suggesting that TMEM63C is not acting as a tether between ER and mitochondria but may have an important role in regulating MERCs function and integrity (Supplementary Fig. 3F). Given this, it may be hypothesized that altered lipid and ion trafficking at MERCs may underlie the organelle morphology abnormalities present in TMEM63C deficient cells.

## Discussion

Here we present data from three unrelated families with HSP, providing compelling evidence that biallelic loss of function sequence alterations in *TMEM63C* cause HSP. All seven affected individuals presented in infancy with consistent clinical features of lower-limb weakness and spasticity typical of HSP. Five additionally present with mild intellectual impairment, which appears to represent a variable clinical outcome associated with biallelic *TMEM63C* variants.

So far, the function of TMEM63C has not been established, nor has it been shown to cause human disease. Previous rat and zebrafish animal model studies of TMEM63C suggested a role in kidney function. Schulz *et al*.^47^ identified *Tmem63c* as a candidate due to its position within the quantitative trait locus region in a strain of hypertensive rats considered to be a suitable model system for investigating the genetic basis of albuminuria. However, no sequence variants were identified in *Tmem63c* in hypertensive rats,^47,48^ and *TMEM63C* expression is absent in human kidneys in the Genotype-Tissue Expression (GTEx) database. Here, we unequivocally identify disruption of TMEM63C function as a cause of neurological disease in humans, consistent with the high expression levels of the *TMEM63* family of molecules in the nervous system.^49^ Further evidence for an important role for TMEM63 family of proteins in neurological function is provided by the previous description of *de novo* heterozygous pathogenic variants in TMEM63A associated with hypomyelinating leukodystrophy, which notably comprises a spasticity component.^50^ Consistent with this, *Tmem63a* knock out mice display gait abnormalities, also indicative of neurological (and motor) impairment.^50^ Similarly, *Tmem63b^−/−^* knock out is associated with an abnormal gait, limb grasping and hyperactivity as well as preweaning lethality in mice.^51,52^ Du *et al*.^31^ also identified a possible role of TMEM63B in hearing by studying *Tmem63b*^−/−^ mice, found to be insensitive to sound stimuli due to severe degeneration of outer hair cells.

To better understand the functional role of TMEM63C, we performed *in cellula* studies to monitor protein subcellular localization, and the impact on both ER and mitochondrial morphologies in two different mammalian cell lines. First, we showed by microscopy analysis that TMEM63C is exclusively localized at the ER, evidenced by its reticular distribution and co-localization with the *bona fide* ER marker, calnexin. In particular, both biochemical and super-resolution microscopy analyses revealed an accumulation of TMEM63C at MERCs. Consistent with a role at MERCs, *TMEM63C* silencing in both HeLa and SH-SY5Y cells resulted in ER and mitochondrial morphological changes primarily characterized by an increase of the balance between ER sheets and tubules, and by mitochondrial elongation and interconnectivity. Mitochondrial hyperfusion is induced by a disruption of the mitochondrial fission–fusion balance and has been identified as a mechanism of defence on cellular stress to enhance cell survival.^39^ The importance of mitochondrial dynamics (including fission, fusion and transport) has been extensively documented to be of critical importance in neuronal development and survival, and alterations in these dynamics are known to contribute to the pathology of several diseases, including neurological disorders.^53^ Although HeLa (derived from cervical cancer cells) and SH-SY5Y (neuroblastoma) cells are well established models for molecular and cell biology studies and provide initial insight into the potential function of TMEM63C at MERCs, both cell lines may be of limited relevance to the study of neuronal physiology in HSP. Therefore, we sought to confirm our findings in more physiologically relevant differentiated and maturated motor neuron-like NSC-34 cells. This again showed that TMEM63C loss leads to a defective mitochondrial morphology network, further emphasizing that altered mitochondrial dynamics potentially underlie the pathological manifestations observed in HSP patients. In this context, as our data suggest that global mitochondrial respiration is not grossly affected, we hypothesize that the mitochondrial elongation observed in TMEM63C-deficient cells may affect organelle motility through the axon resulting in neuronal bioenergetic defects when a specific or rapid production of ATP is required at specific cellular subdomains.^54^ Moreover, enlargement of mitochondria may negatively affect the respiration capacity and result in mitochondria that are more resistant to selective degradation by mitophagy.^55^ This autophagic degradation defect may impair the proper turnover of the organelle, leading to the accumulation of damaged and dysfunctional mitochondria, which may contribute to axonal degeneration.^56^ Similarly, it has been suggested that the increased ratio of ER sheet to tubules may underlie the partial loss of the organelle in the distal motor axons, as described with receptor accessory protein 1 (*REEP1*) gene variants associated with HSP.^36^ Indeed, the reduction of ER membranes in distal axons may not only affect the lipid composition of the different neuronal membranes and decrease membrane contact sites between the ER and other organelles, but also alter the local generation and propagation of Ca^2+^ fluxes that is critically important for axon function.^57^

Together, these data support an important role for TMEM63C in maintaining ER-mitochondrial organelle homeostasis and structure, likely to underlie HSP pathological outcomes. Maintained MERCs integrity is known to be of critical importance for a number of subcellular processes crucial for neuronal health, including lipid and calcium homeostasis.^58^ Our data show that *TMEM63C* silencing alters organelle integrity of both mitochondria and ER, reflecting a potential role for this protein in the maintenance of these structures. Notably there is increasing evidence linking the role of HSP-associated genes with lipid homeostasis, including oxysterol-cholesterol and phosphatidylethanolamine metabolic pathways,^2,59^ mediated in part through MERCs.^2^ The molecular role of TMEM63C proposed here is thus consistent with a potential role in lipid (and other) metabolic processes mediated through MERCs, and in the regulation of organelle morphology and cellular homeostasis. Further work is required to understand the precise cellular role of TMEM63C, and the specific pathomolecular outcomes associated with pathogenic *TMEM63C* variants. One limitation of the current study has been that we have been unable to obtain patient-derived fibroblasts to investigate the effects of *TMEM63C* gene variants in the most appropriate cellular models. Indeed, while our data show clear parity across a range of cell lines including differentiated and maturated motor neuron-like NSC-34 cells, they fail to completely mimic the selective neuronal vulnerability observed in HSP and therefore fully reveal the neuropathological basis of disease in patients. In future, it will be valuable to perform similar studies in active motor neurons reprogrammed from patient-derived fibroblasts using induced-pluripotent stem cell (iPSC) technology, to corroborate the role of TMEM63C in maintaining organelle morphology, but also to more fully reveal the HSP neuro-pathomechanism. Such endeavours will further increase our understanding of how deficits in ER—mitochondrial connectivity and function may lead to motor neuron degenerative disease, paving the way for targeted therapeutics.

## Supplementary Material

awac123_Supplementary_Data

## Abbreviations

ER =: endoplasmic reticulum
HSP =: hereditary spastic paraplegia
MAM =: mitochondria-associated membranes
MERCs =: mitochondria-ER contact sites
siNT =: non-targeting small interfering RNA
siRNA =: small interfering RNA
SNP =: single nucleotide polymorphism
WES =: whole exome sequencing
