# Correction to: TMEM63C mutations cause mitochondrial morphology defects and underlie hereditary spastic paraplegia

**DOI:** 10.1093/brain/awac254

**Published:** 2022-09-09

Luis Carlos Tábara, Fatema Al-Salmi, Reza Maroofian, Amna Mohammed Al-Futaisi, Fathiya Al-Murshedi, Joanna Kennedy, Jacob O. Day, Thomas Courtin, Aisha Al-Khayat, Hamid Galedari, Neda Mazaheri, Margherita Protasoni, Mark Johnson, Joseph S. Leslie, Claire G. Salter, Lettie E. Rawlins, James Fasham, Almundher Al-Maawali, Nikol Voutsina, Perrine Charles, Laura Harrold, Boris Keren, Edmund R. S. Kunji, Barbara Vona, Gholamreza Jelodar, Alireza Sedaghat, Gholamreza Shariati, Henry Houlden, Andrew H. Crosby, Julien Prudent, Emma L. Baple. TMEM63C mutations cause mitochondrial morphology defects and underlie hereditary spastic paraplegia. *Brain*. 2022;145(9):3095–3107. https://doi.org/10.1093/brain/awac123

The authors apologize for errors in the labelling of p.(Asn547Lysfs*42) and p.(Trp406Glyfs*26) variants in Fig. 1A and D and Table 1. These have been corrected.

