# Supplementary material for: TMEM63C mutations cause mitochondrial morphology defects and underlie hereditary spastic paraplegia
Source: Brain. 2022 Jun 18;145(9):3095–107. doi: 10.1093/brain/awac123 (PMC9473353; doi:10.1093/brain/awac123)
Supplement: awac123_Supplementary_Data [file awac123_supplementary_data.zip › brain-2021-01369-File006.pdf]

TMEM63C mutations cause mitochondrial morphology defects and underlie hereditary spastic paraplegia

Supplementary Material

Luis-Carlos Tábara[1,†], Fatema Al-Salmi[2,†], Reza Maroofian[3,†], Amna Mohammed Al-Futaisi[4], Fathiya Al-Murshedi[4], Joanna Kennedy[2,5], Jacob O Day[2,6], Thomas Courtin[7], Aisha Al-Khayat[8], Hamid Galedari[9], Neda Mazaheri[9], Margherita Protasoni[1], Mark Johnson[1], Joseph Leslie[2], Claire G Salter[2], Lettie E Rawlins[2,10], James Fasham[2,10], Almundher Al-Maawali[4], Nikol Voutsina[2], Perrine Charles[7], Laura Harrold[2], Boris Keren[7], Edmund RS Kunji[1], Barbara Vona[11], Gholamreza Jelodar[12], Alireza Sedaghat[13], Gholamreza Shariati[14], Henry Houlden[3], Andrew H Crosby[2#*], Julien Prudent[1#*], Emma L Baple[2,10#*]

[†] **These authors contributed equally to this work.**

[#] **These authors contributed equally to this work.**

*** Corresponding author**

1. Medical Research Council Mitochondrial Biology Unit, University of Cambridge, Cambridge, CB2 0XY, UK
2. Level 4, RILD Wellcome Wolfson Medical Research Centre, RD&E (Wonford) NHS Foundation Trust, University of Exeter Medical School, Barrack Road, Exeter, EX2 5DW, UK
3. UCL Queen Square Institute of Neurology, University College London, WC1E 6BT, UK
4. Genetic and Developmental Medicine Clinic, Department of Genetics, College of Medicine and Health Sciences, Sultan Qaboos University Hospital, Muscat 123, Oman
5. Clinical Genetics, University Hospitals Bristol, Southwell St, Bristol, BS2 8EG, UK
6. Faculty of Health, University of Plymouth, Plymouth, PL4 8AA, UK
7. Département de génétique, Hôpital Pitié-Salpêtrière, Assistance Publique-Hôpitaux de Paris, 75019, Paris, Sorbonne Université, France
8. Department of Biology, College of Science, Sultan Qaboos University, Muscat, Oman

9. Department of Genetics, Faculty of Science, Shahid Chamran University of Ahvaz, Ahvaz, Iran
10. Peninsula Clinical Genetics Service, Royal Devon & Exeter Hospital (Heavitree), Gladstone Road, Exeter, EX1 2ED, UK
11. Department of Otolaryngology-Head and Neck Surgery, Tübingen Hearing Research Centre, Eberhard Karls University Tübingen, Tübingen, Germany
12. Pediatric Neurology, Ahvaz Jundishapur University of Medical Sciences, Ahvaz, Iran
13. Health Research Institute, Diabetes Research Center, Ahvaz Jundishapur University of Medical Sciences, Ahvaz, Iran
14. Department of Medical Genetic, Faculty of Medicine, Ahvaz Jundishapur, University of Medical Sciences, Ahvaz, Iran

Correspondence to:

Professor Andrew Crosby

RILD Wellcome Wolfson Centre, Royal Devon & Exeter NHS Foundation Trust, Barrack Road, Exeter, EX2 5DW, UK

Email: A.H.Crosby@exeter.ac.uk

Dr. Julien Prudent

MRC Mitochondrial Biology Unit, University of Cambridge, The Keith Peters building, Cambridge Biomedical Campus, Hills road, Cambridge, CB2 0ZY, UK.

Email: julien.prudent@mrc-mbu.cam.ac.uk

Dr. Emma Baple

RILD Wellcome Wolfson Centre, Royal Devon & Exeter NHS Foundation Trust, Barrack Road, Exeter, EX2 5DW, UK

Email: E.Baple@exeter.ac.uk

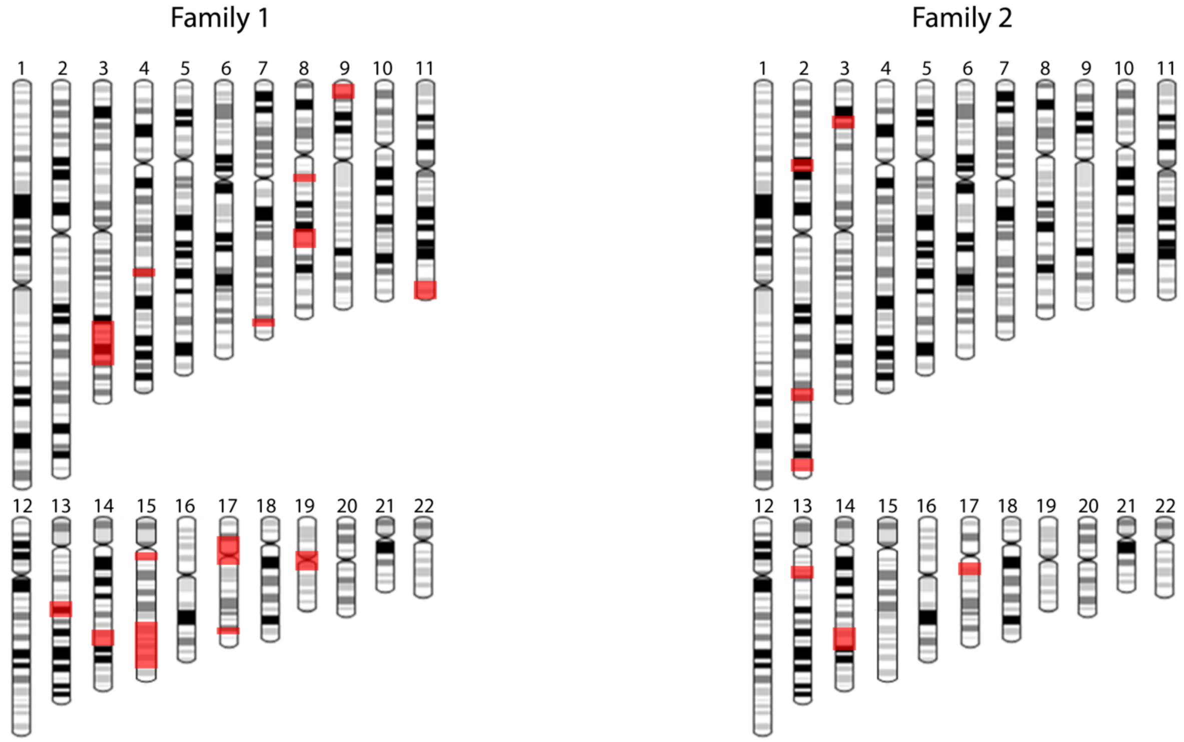

Supplementary Figure S1. Genome-wide homozygosity mapping in families 1 and 2 identifying homozygous haplotypes common to affected individuals in each family (Individuals IV:2 and IV:8 in family 1 and individuals V:I, V:II, VI:I, VI:II in family 2) shown as red blocks on the chromosomal karyotype schematic. The *TMEM63C* gene is located in a ~10.1Mb homozygous region on chromosome 14q24.3 common to both affected individuals in family 1, and in a ~9.2Mb homozygous region on chromosome 14q24.3 common to all four affected individuals in family 2.

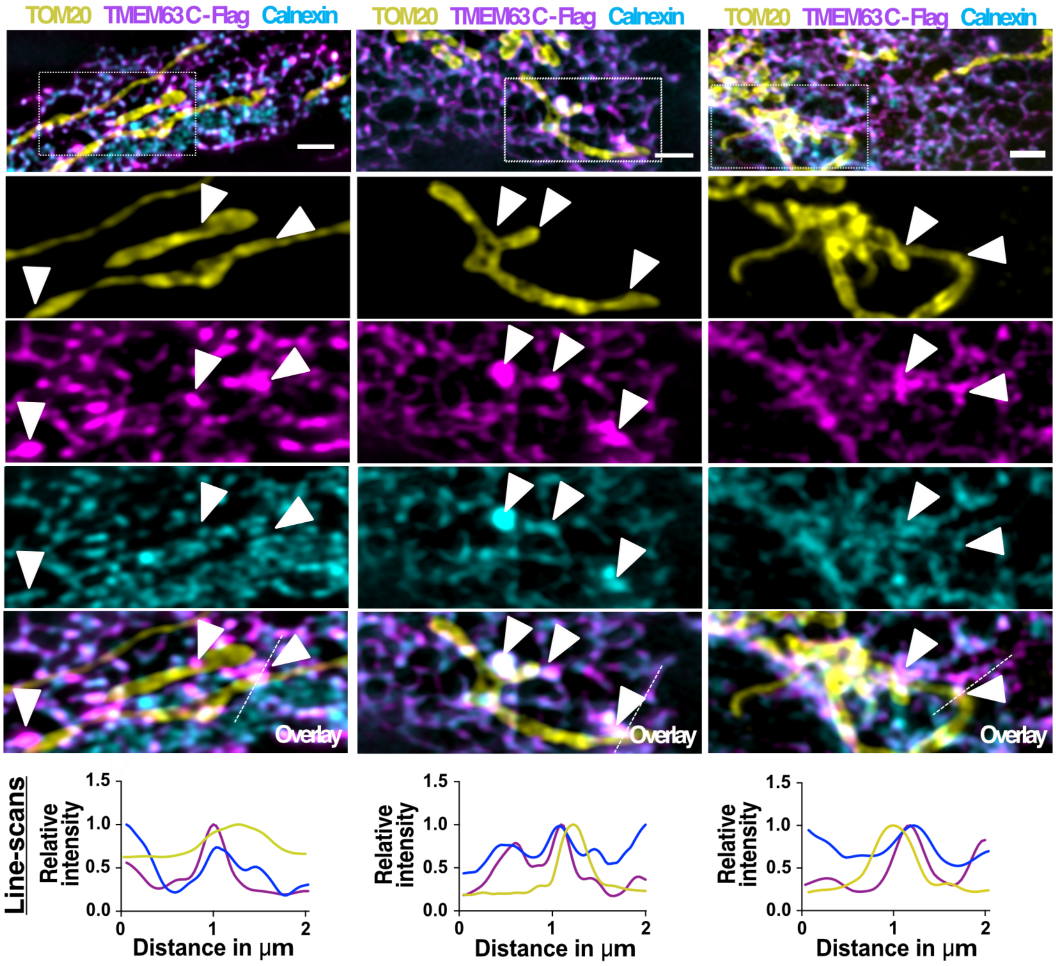

Supplementary Figure S2. Other examples of representative super-resolution N-Structured Illumination Microscopy (N-SIM) images of HeLa cells expressing TMEM63C-Flag, showing TMEM63C-Flag *foci* accumulation at mitochondria-endoplasmic reticulum (ER) contact sites (white arrows). Flag, mitochondria and ER were labelled with anti-Flag, anti-TOM20 and anti-calnexin antibodies, respectively. 2 μm line-scan analysis (bottom panels) of relative fluorescence intensity from the dashed line are shown. Scale bars: 2 μm.

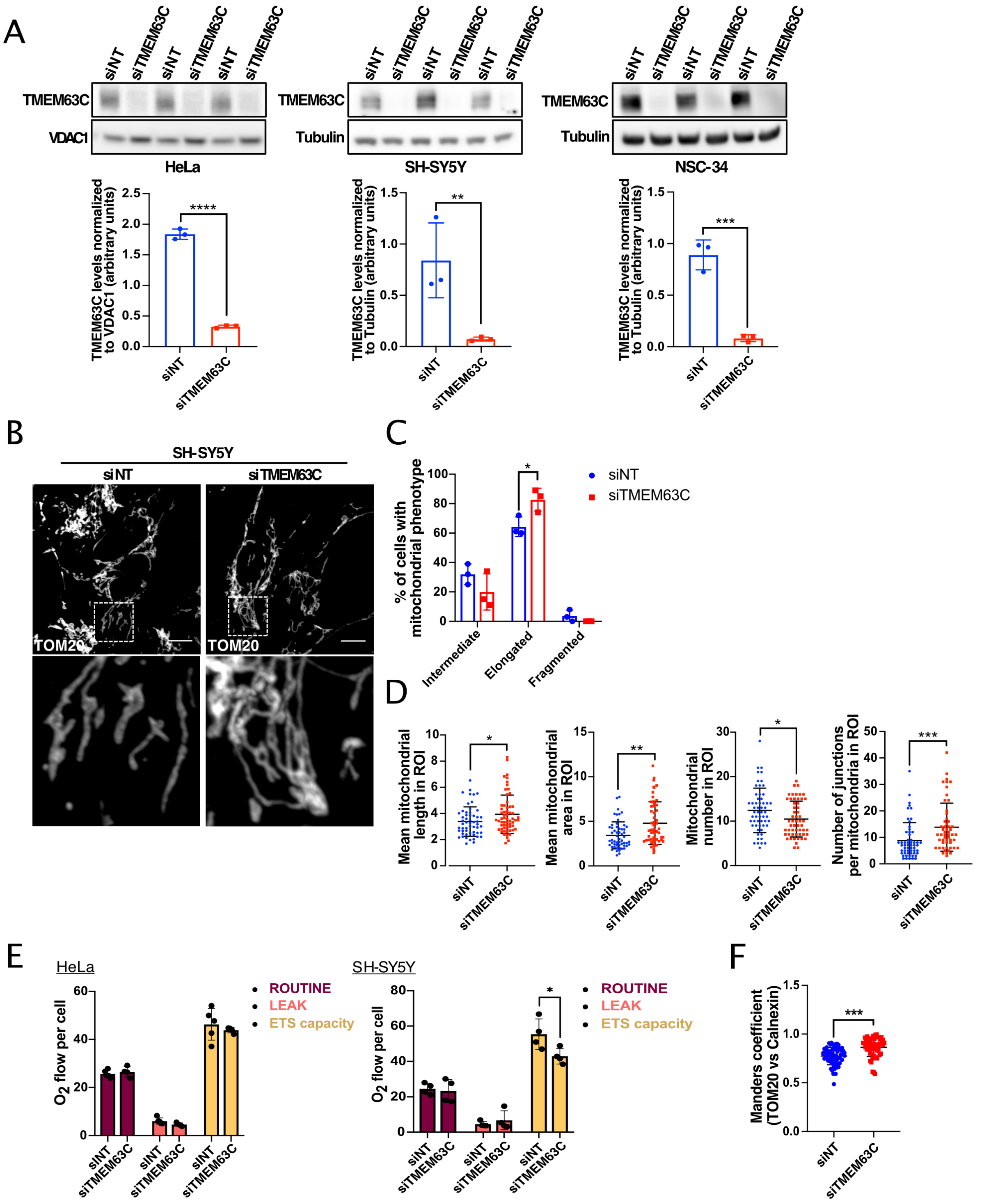

Supplementary Figure S3. (A) Immunoblot analysis and associated quantifications showing the efficiency of *TMEM63C* siRNAs in the different cell lines used in this study (HeLa, SH-SY5Y and NSC-34 cells). VDAC1 and Tubulin were used as loading controls to normalize the protein levels of TMEM63C. Three independent experiments are shown. **(B)** Representative confocal images of mitochondrial morphology of control (siNT) and *TMEM63C* (siTMEM63C) silenced SH-SY5Y cells. Mitochondria were labelled using an

anti-TOM20 antibody. Scale bars: 10 μm. **(C)** Quantification of mitochondrial morphology related to (B). **(D)** Quantification of different mitochondrial morphology parameters including mean mitochondrial length and area, mitochondrial number and mitochondrial branching measured by mitochondrial junction number, per region of interest (ROI) of 225 μm^2, related to (B). **(E)** High-resolution respirometry analyses performed in *TMEM63C* silenced intact HeLa (left panel) and SH-SY5Y (right panel) cells in an Oroboros instrument, compared to control cells. ROUTINE: cellular basal oxygen consumption rate (in pmol O_2/sec) per million cells in culture DMEM medium. LEAK: non-phosphorylating respiration in the presence of the ATP synthase inhibitor oligomycin. ETS capacity: maximal capacity of the electron transfer system in the presence of the uncoupler CCCP. Background was calculated after inhibiting complex I and complex III with rotenone and antimycin A, respectively. **(F)** Quantification of endoplasmic reticulum (ER) and mitochondria overlapping areas using Mander's coefficient related to Figure 3A. All data are shown as mean ± SD of at least three independent experiments. For (A), unpaired t-test (two-tailed) was used; for (C) and (E), two-way ANOVA and Tukey's multiple-comparisons tests was used; for (D) and (F), Mann-Whitney U-test (two-tailed) was used; $*P < 0.05$, $**P < 0.01$, $***P < 0.001$, $****P < 0.0001$.

FIGURE 2B

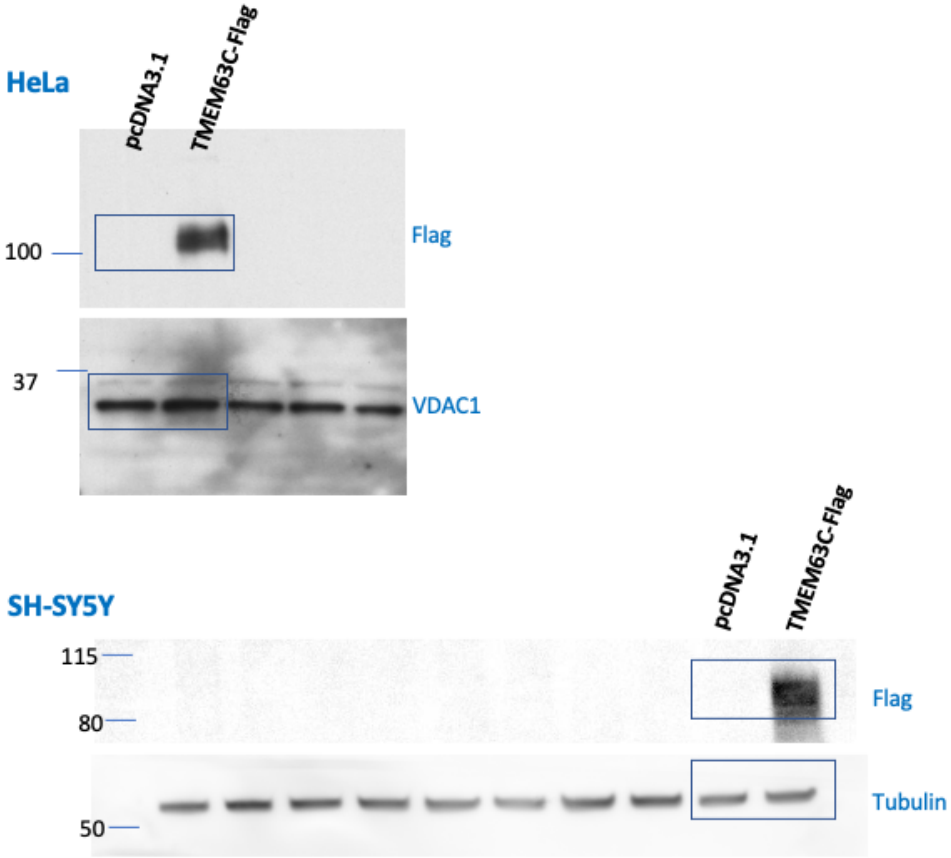
HeLa
pcDNA3.1
TMEM63C-Flag
100
Flag
37
VDAC1
SH-SY5Y
pcDNA3.1
TMEM63C-Flag
115
80
Flag
50
Tubulin

FRACTIONATION: ACSL4, VDAC1

FIGURE 2D

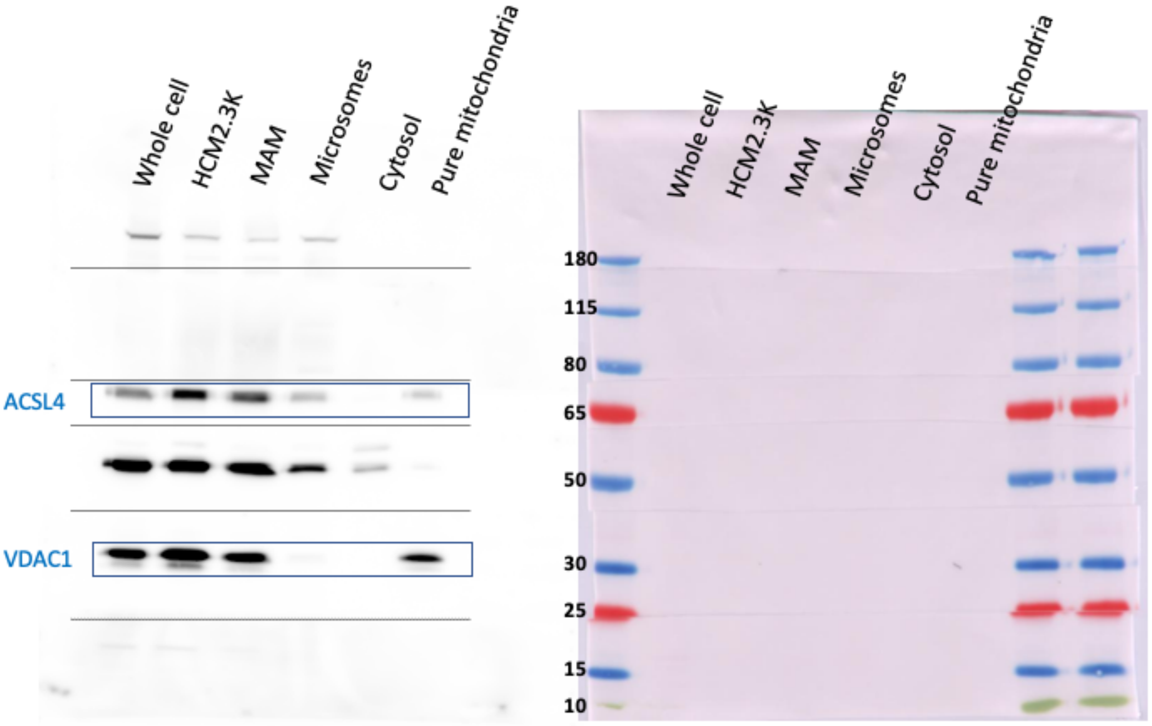

FRACTIONATION: VAPB

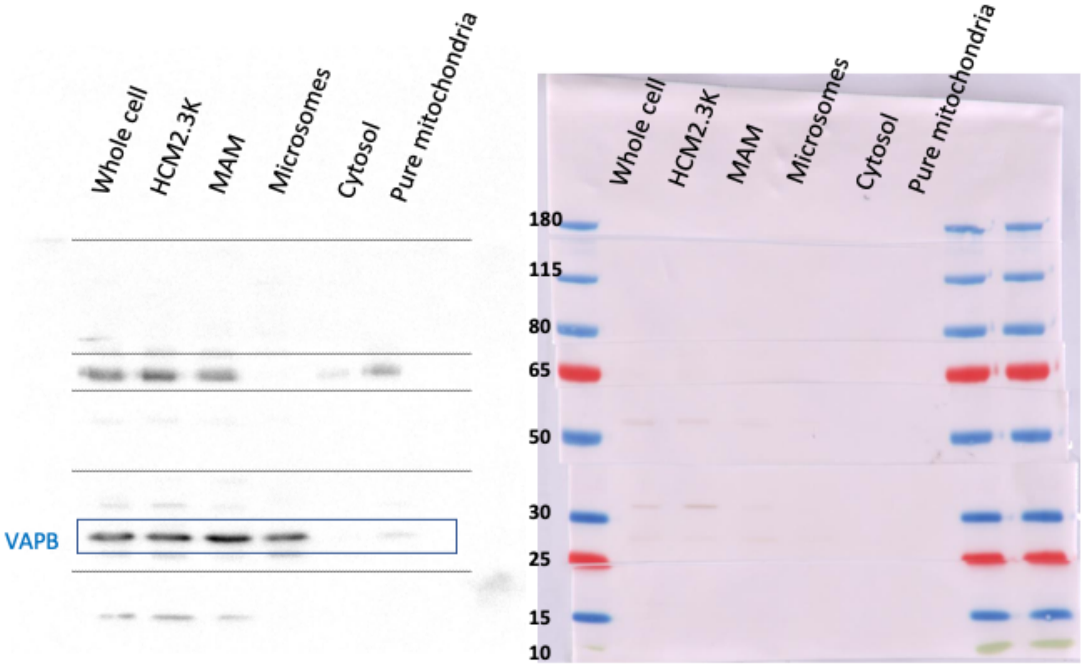

FRACTIONATION: CALNEXIN

FIGURE 2D

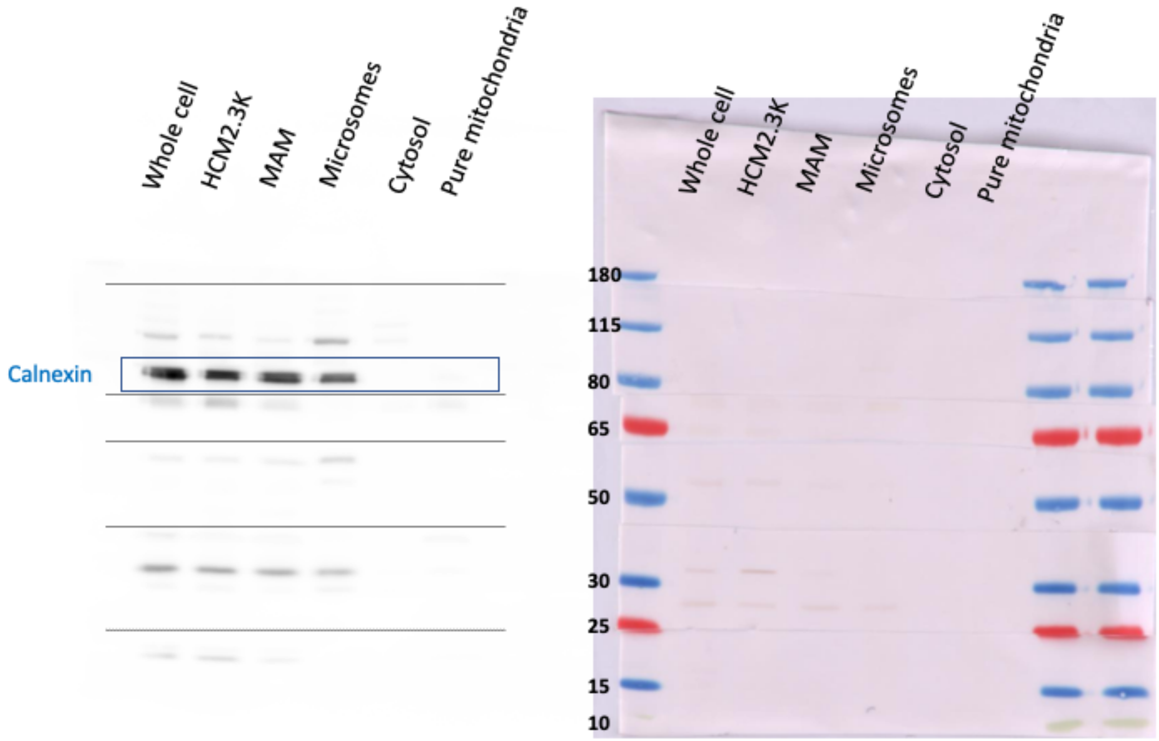

FRACTIONATION: TMEM63C

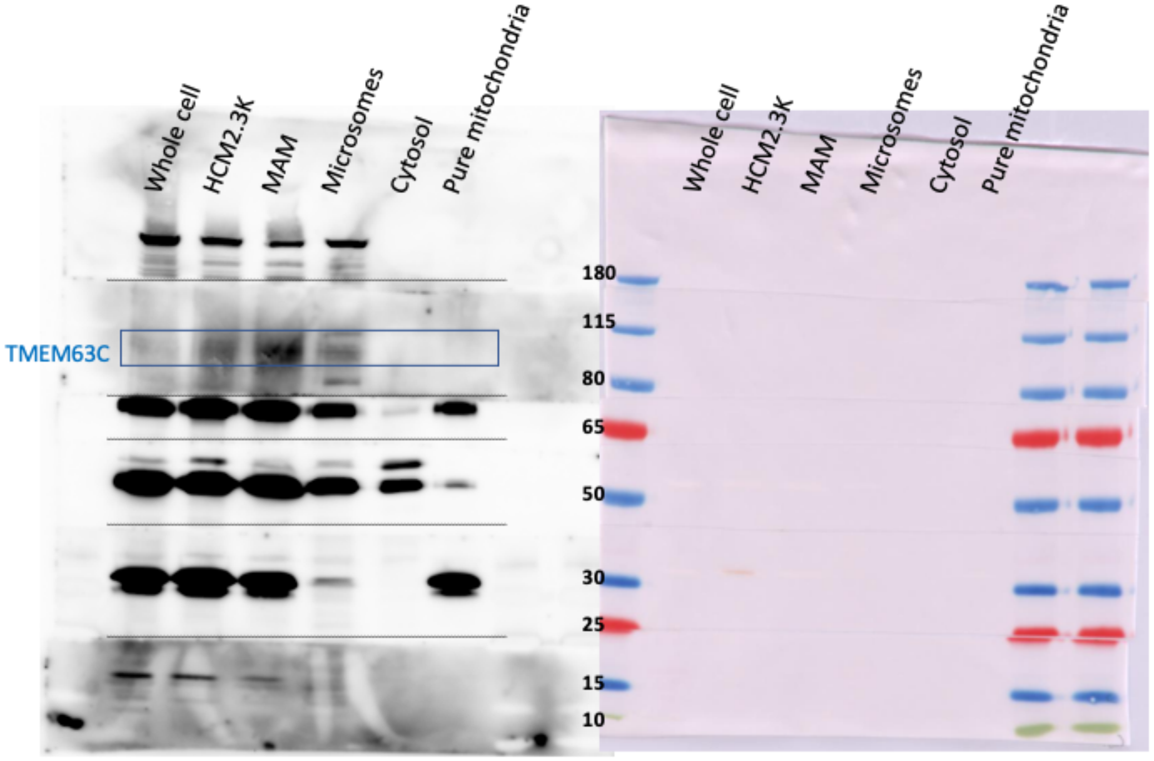

FRACTIONATION: PEX14

FIGURE 2D

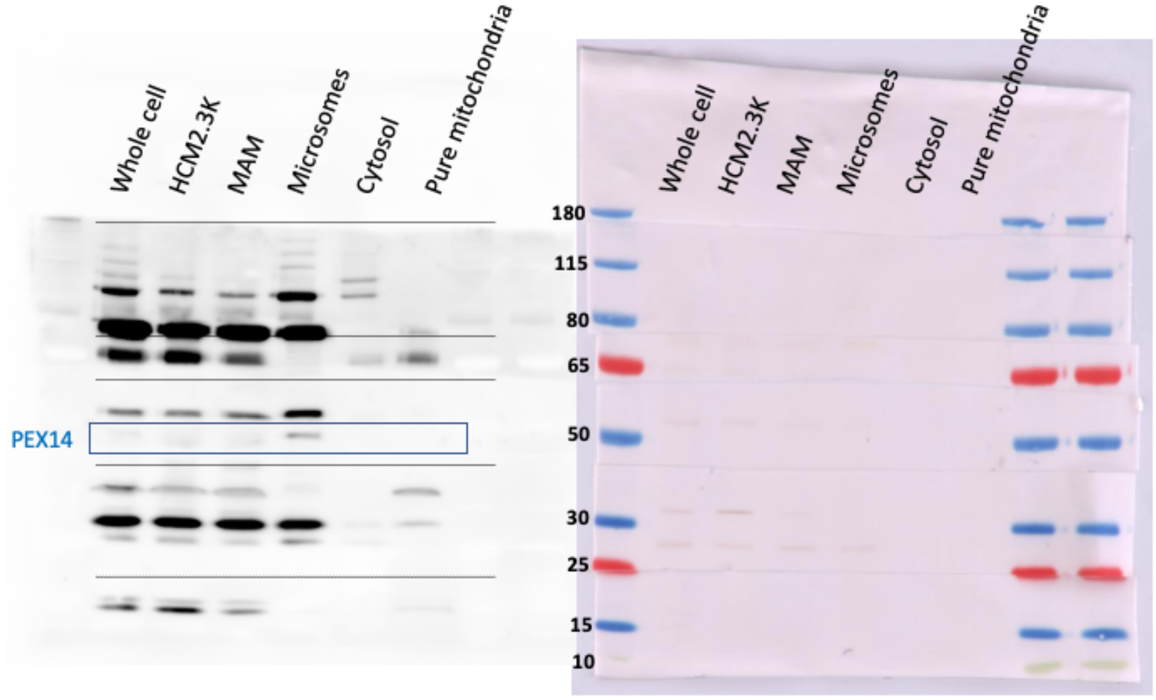

Higher exposure:

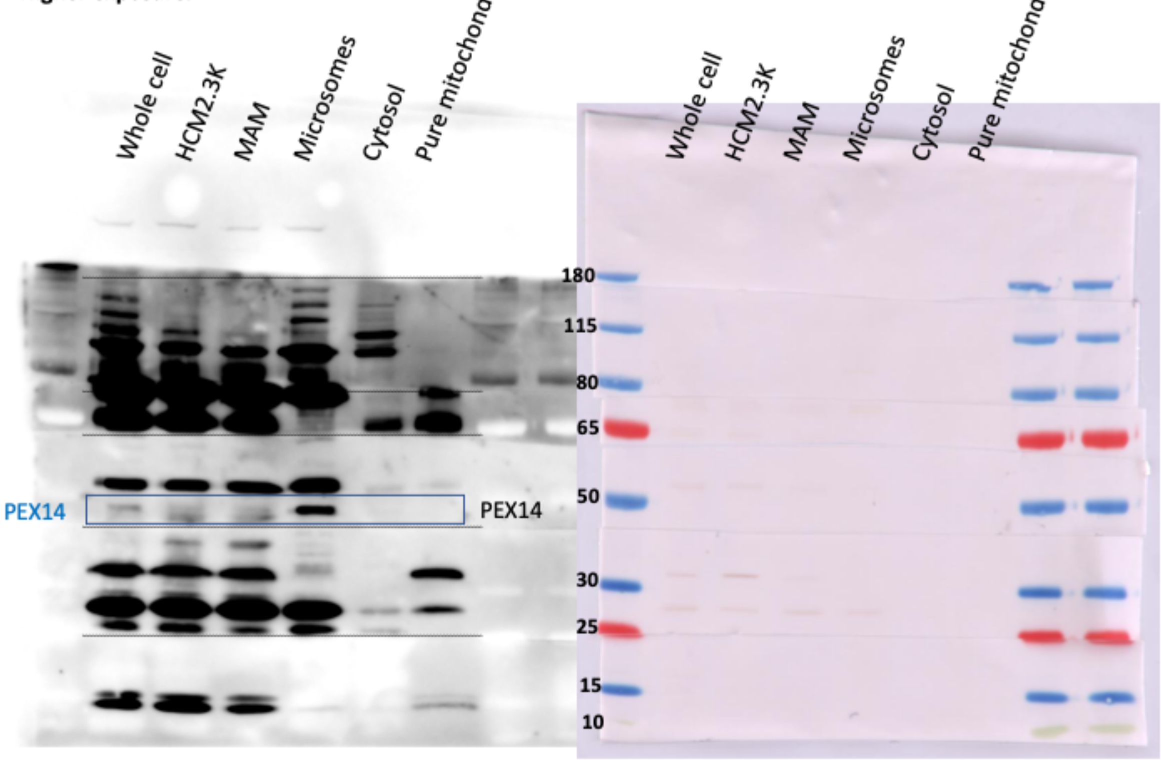

FRACTIONATION: TUBULIN

FIGURE 2D

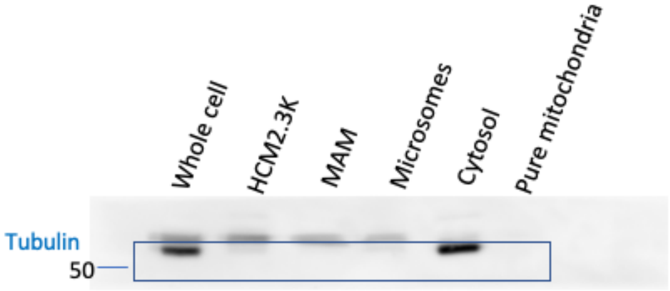

FIGURE S3A

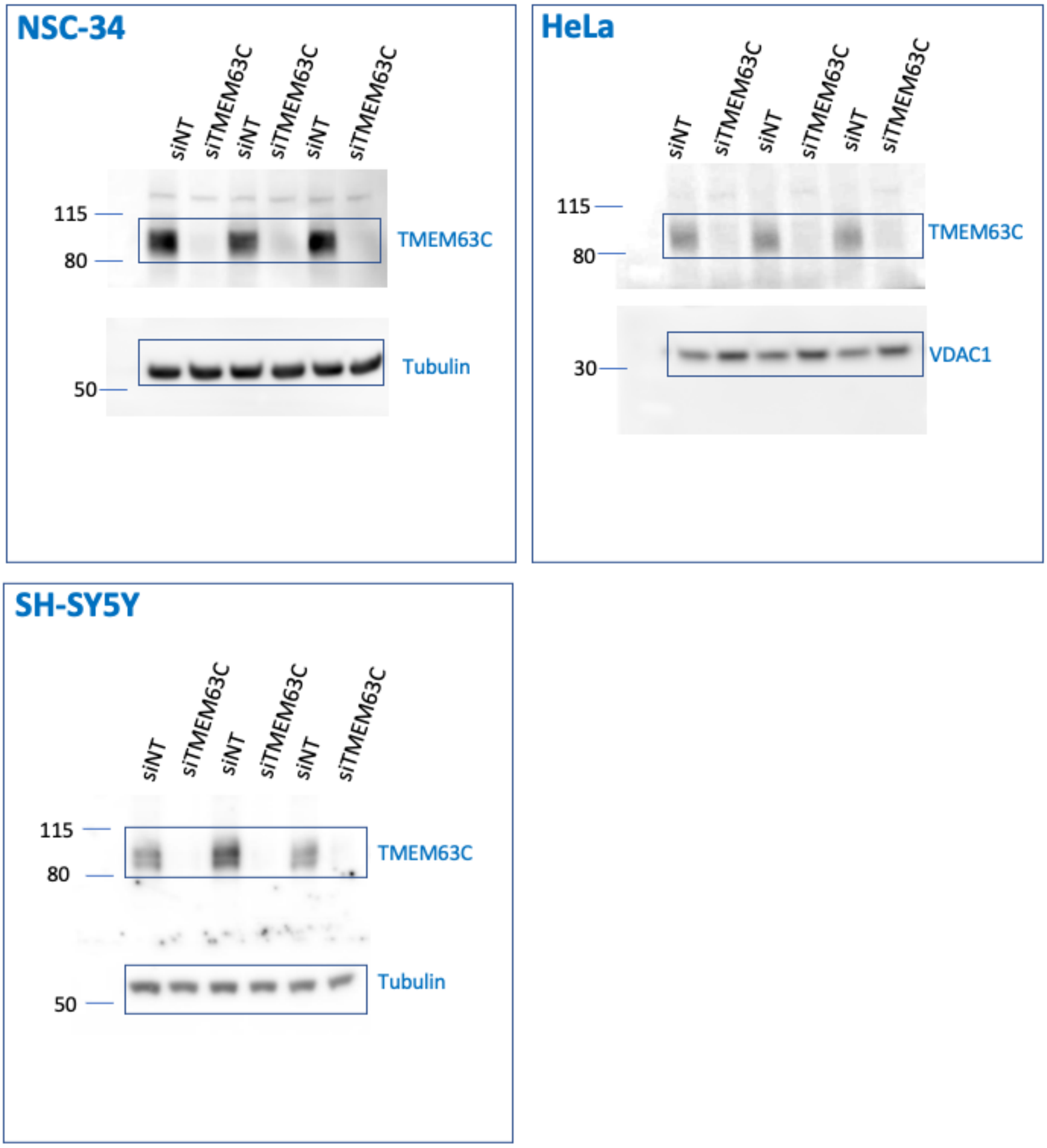

Supplementary Figure S4. Uncropped scans for the immunoblots presented in the figures.

Supplementary Table 1. Genomic variants identified in each family that could not be excluded by co-segregation analysis

Family	Gene	Zygosity	GRChr37:g	c.Nomen	p.Nomen	gnomAD v2.1.1 MAF	*In silico* predictions	
							Polyphen	SIFT
1	COBL	Hom	Chr7: g.51251809C>T	NM_015198.4: c.773G>A	NM_015198.4: p.(Ser258Asn)	5.76 x 10^{-6}	Possibly damaging	Tolerated
	GRID2IP	Hom	Chr7: g.6548686C>T	NM_001145118.1: c.2030G>A	NM_001145118.1: p.(Arg677His)	2.37 x 10^{-5}	Benign	Damaging
	RAVER2	Hom	Chr1: g.65243641A>G	NM_018211.3: c.652A>G	NM_018211.3: p.(Met218Val)	Not present	Probably damaging	Tolerated
	KIAA1755	Hom	Chr20: g.36874513C>T	NM_001348708.1: c.19G>A	NM_001348708.1: p.(Asp7Asn)	Not present	Probably damaging	Damaging
	LSMEM1	Hom	Chr7: g.112129991C>T	NM_001134468.1: c.383C>T	NM_001134468.1: p.(Ser128Phe)	9.62 x 10^{-4}	Benign	Damaging
	ARIH1	Hom	Chr15: g.72837255T>A	NM_005744.3: c.538T>A	NM_005744.3: p.(Ser180Thr)	1.59 x 10^{-5}	Benign	Tolerated
	MIEF2	Hom	Chr17: g.18166069G>T	NM_148886.1: c.68G>T	NM_148886.1: p.(Arg23Leu)	Not present	Benign	Damaging
	RBBP6	C Het*	Chr16: g.24560283C>T	NM_006910.4: c.284C>T	NM_006910.4: p.(Ala95Val)	4.31 x 10^{-5}	Benign	Tolerated
	RBBP6	C Het*	Chr16: g.24573280T>C	NM_006910.4: c.1087T>C	NM_006910.4: p.(Ser363Pro)	2.51 x 10^{-4}	Benign	Tolerated
	ACACB	C Het*	Chr12: g.109609695C>G	NM_001093.3: c.1011C>G	NM_001093.3: p.(Asp337Glu)	1.59 x 10^{-5}	Probably damaging	Tolerated
	ACACB	C Het*	Chr12: g.109678925C>T	NM_001093.3: c.4861C>T	NM_001093.3: p.(Arg1621Trp)	3.98 x 10^{-6}	Probably damaging	Damaging
2	No additional variants identified after co-segregation							
3	Not available							
